# Marine Invertebrate-Inspired Thermal Management: Functional Materials, Structural Architectures, and Integrated Systems

**DOI:** 10.3390/biomimetics11060373

**Published:** 2026-05-27

**Authors:** Hoejin Jung, Inhye Shin, Sunwoo Kim, Sieun Jung, Jaeik Kim, Won-gyu Bae

**Affiliations:** 1 Department of Electrical Engineering, Soongsil University, Seoul 06978, Republic of Korea; wjdghlwls1234@soongsil.ac.kr (H.J.); sw3455@soongsil.ac.kr (S.K.); kimij1008@soongsil.ac.kr (J.K.); 2 Department of Chemical Engineering, Soongsil University, Seoul 06978, Republic of Korea; inhye0531@soongsil.ac.kr (I.S.); se5818@soongsil.ac.kr (S.J.)

**Keywords:** marine invertebrates, bio-inspired thermal engineering, thermal management, heat transfer, thermal functional materials, thermal management systems

## Abstract

Marine invertebrates exhibit diverse thermoregulatory capabilities enabled by hierarchical architectures, porous skeletal frameworks, and adaptive interfaces. These biological features provide engineering cues for controlling heat conduction, convection, and radiation, particularly when lightweight and multifunctional thermal designs are required. This review surveys marine-invertebrate-inspired thermal management from an engineering perspective and synthesizes biological structure–function relationships into transferable design concepts. Literature was collected from Scopus, Web of Science, and Google Scholar. Studies were included if they (i) explicitly referenced marine invertebrate morphology, structural organization, interfacial behavior, or adaptive mechanisms and (ii) quantitatively reported thermal metrics such as thermal conductivity, heat capacity/latent heat, heat dissipation performance, or temperature modulation. To maintain biological scope while enabling cross-comparison, the review is organized across major marine invertebrate phyla frequently used in bioinspired engineering—Mollusca, Porifera, Cnidaria, Echinodermata, and Arthropoda—and the engineering literature is classified into three categories: (A) bio-inspired functional materials for thermal transport or optical–thermal control; (B) bio-inspired structural architectures that guide heat flow via hierarchical or porous geometries; and (C) integrated thermal management systems that couple multiple mechanisms at the device or system scale. Across these categories, the reviewed studies demonstrate promising routes toward electronics cooling and aerospace thermal protection. Remaining challenges include scalable fabrication over large areas, flow uniformity in microchannel-based platforms, and long-term reliability under combined pressure, salinity, and thermal cycling.

## 1. Introduction

Thermal management is a central engineering consideration in systems where heat transport, dissipation, or insulation directly affects performance and reliability. This is particularly evident in electronic devices, aerospace components, and compact thermal systems [1,2]. In such applications, conventional approaches are often based on homogeneous materials or simplified geometries. These limitations motivate alternative design strategies that enable more flexible control of heat conduction, convection, and radiation. In this context, bio-inspired approaches have been explored as a means of introducing structural hierarchy and heterogeneous interfaces into thermal management design. Such approaches also enable geometry-driven control of heat transfer. Studies applying the structural and interfacial characteristics of marine invertebrates to thermal management design have been reported in various forms [3,4]. These studies draw inspiration from shells, skeletal frameworks, surface architectures, and interfacial features of marine invertebrates. Importantly, these features can be translated into engineered materials, structures, and devices without relying on organism-specific functionality. A growing body of work has leveraged these motifs to address distinct heat-transfer objectives. Reported examples include hierarchical porous architectures that reduce effective thermal conductivity, geometry-engineered structures that modify convective heat-transfer behavior, and structured or adaptive surfaces that regulate optical–thermal interactions through solar absorption and infrared emission. Several studies further extend these concepts to system-level implementations, such as thermal diffusion layers, microchannel heat exchangers, and adaptive thermal interfaces. However, the literature remains fragmented: studies are often presented as case-specific demonstrations tied to particular organisms or isolated functions, while cross-comparison is hindered by inconsistent organization and, in many cases, nonuniform reporting of dominant heat-transfer modes and quantitative thermal metrics. Existing reviews have addressed biomimetic thermal materials or bio-inspired heat-transfer structures in broader scopes. Nevertheless, an engineering-oriented synthesis that consolidates marine invertebrate–inspired strategies and systematically links biological design motifs to quantitative thermal metrics within a unified comparative framework remains limited. Moreover, practical constraints critical for translation—scalable fabrication, flow uniformity in microchannel-based platforms, and long-term reliability under coupled pressure, salinity, and thermal cycling—are rarely discussed in an integrated manner. To address these issues, this review organizes marine invertebrate–inspired thermal management across major marine invertebrate lineages while classifying the engineering literature into three implementation levels: (A) bio-inspired functional materials, (B) bio-inspired structural architectures, and (C) integrated thermal management systems. Within each category, studies are compared using reported thermal metrics—such as thermal conductivity, heat capacity/latent heat, heat dissipation performance, and temperature modulation—and interpreted in terms of the dominant heat-transfer mechanisms they manipulate (conduction, convection, and radiation). By structuring dispersed case studies within a consistent engineering frame, this review clarifies research trends, identifies technical limitations, and outlines design opportunities for practical thermal management technologies inspired by marine invertebrates.

### 1.1. Review Methodology

This review was conducted through a structured literature survey using Scopus, Web of Science, and Google Scholar. The search was performed using combinations of keywords related to marine invertebrates, bioinspired thermal management, and heat-transfer performance, including “marine invertebrate”, “bioinspired thermal management”, “mollusk-inspired”, “cuttlebone-inspired”, “nacre-inspired”, “sponge-inspired”, “coral-inspired”, “thermal conductivity”, “thermal insulation”, “radiative cooling”, “heat dissipation”, and “temperature modulation”. Studies published up to 2025 were considered. Studies were included when they met two main criteria: (i) the work explicitly referenced marine invertebrate morphology, hierarchical structure, interfacial behavior, or adaptive mechanisms, and (ii) the work reported thermal-management-relevant outcomes, such as thermal conductivity, heat capacity or latent heat, radiative cooling performance, heat dissipation, thermal insulation, temperature reduction, or flow-related heat-transfer behavior. Studies were excluded when they focused solely on biological taxonomy without engineering translation, lacked thermal management relevance, did not report quantitative or clearly interpretable performance outcomes, or were based on non-marine or terrestrial biological models outside the scope of this review. The selected studies were analyzed by linking biological design motifs to dominant heat-transfer mechanisms, including conduction, convection, radiation, and interfacial heat transfer. They were further classified into three engineering categories: bio-inspired functional materials, bio-inspired structural architectures, and integrated thermal management systems. This classification was used to compare the reviewed studies in terms of biological inspiration, engineering implementation, reported thermal outcomes, and practical limitations.

### 1.2. Comparative Analysis with Existing Literature

Although bioinspired thermal-management studies have extensively explored terrestrial organisms and photonic biological structures, marine invertebrates remain comparatively less systematized as a source of thermal-management design principles. Unlike many previously emphasized biological models, marine invertebrates integrate shell-derived geometries, hierarchical porous skeletons, adaptive optical skins, and organic–inorganic layered architectures within fluid-rich and environmentally complex marine conditions. These features make them particularly suitable for constructing an integrated biomimetic framework that connects heat dissipation, optical regulation, heat–mass transfer, and thermally functional composite design [5]. The structural features of marine invertebrates are closely related to several design axes currently emphasized in thermal-management engineering. Molluscan shells, including limpet- and gastropod-like geometries, provide morphology-driven inspiration for compact heat-dissipation structures because their conical, spiral, and helicoidal forms are associated with surface-area utilization, flow-path modulation, thermal resistance, Nusselt number, and fin efficiency. Recent studies on pin-fin heat sinks, including conical and inverted-conical geometries [6], further highlight geometric optimization as a key strategy for electronic cooling systems. Cephalopods, such as squids, octopuses, and cuttlefish, provide another important model by linking adaptive optical modulation with thermal-regulation concepts. Their chromatophore-, iridophore-, leucophore-, and papillae-based skin systems enable dynamic control of absorption, reflection, transmission, scattering, color, and texture, inspiring stimuli-responsive optical and thermal-regulation materials [7]. Squid-skin-inspired thermoregulatory materials further demonstrate the relevance of cephalopod mechanisms to tunable infrared transmittance and heat-flux regulation [8]. Coral and nacre expand this platform from optical and geometric design to porous heat–mass transfer and composite thermal management. Coral skeletons possess hierarchical pores and interconnected channels that support water transport, solar absorption, vapor escape, heat localization, and optical scattering; accordingly, coral skeleton-based and coral-inspired structures have been applied to interfacial solar evaporation and passive radiative cooling [9]. Meanwhile, nacre-inspired brick-and-mortar architectures provide design principles for integrating mechanical robustness with thermal insulation, anisotropic heat conduction, leakage suppression, and phase-change thermal buffering [10]. Taken together, marine invertebrates provide an integrated biomimetic platform that links geometry-controlled heat dissipation, adaptive optical and infrared regulation, porous heat–mass transfer, passive radiative cooling, anisotropic thermal pathways, and mechanically reinforced thermal composites. Therefore, this review focuses on marine invertebrate-inspired thermal-management strategies at the material, structural, and system levels, emphasizing their potential to expand the design space for next-generation thermal-management technologies.

## 2. Thermal Management Strategies Inspired by Mollusca

**Figure 1:**
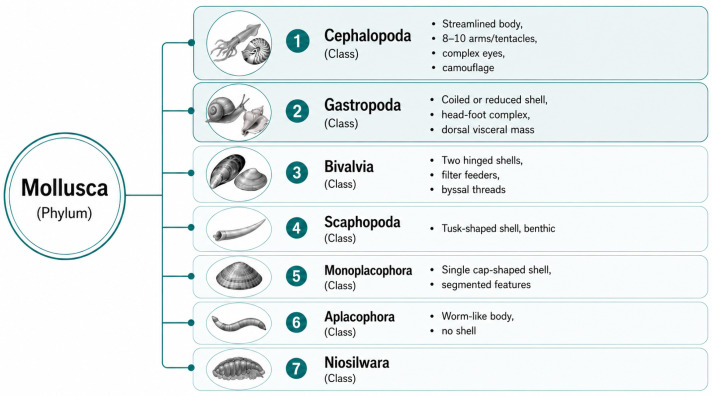
Overview of Mollusca’s major classes and representative morphological features.

As shown in Figure 1, Mollusca is a highly diverse metazoan phylum with an evolutionary history extending at least to the Early Cambrian [11,12], and this diversity is linked by conserved developmental origins, mantle-derived dorsal organization [12,13], a shared soft body plan [14], and structural diversification within a monophyletic lineage [15]. This structural diversity provides a basis for thermal-management design because molluscan systems integrate adaptive soft tissues, biomineralized shells, hierarchical interfaces, and porous or layered architectures. Squid-derived systems support dynamic optical–thermal regulation [16], cuttlebone-derived porous structures support insulation-oriented design [17], and octopus-inspired systems enable adaptive interfacial heat control [18,19]. Gastropod nacre-inspired structures provide layered mechanical and multifunctional design templates [20], while BNNS/epoxy nacre-like composites demonstrate directional thermal-conductive pathways [21]. High-spired shells provide spiral templates for vibration attenuation and thermo-mechanical protection [22]. Bivalve-derived systems further support wet-stable interfacial heat transfer [23,24]. Accordingly, Cephalopoda, Gastropoda, and Bivalvia are emphasized as representative molluscan platforms for thermal management.

### 2.1. Cephalopod-Inspired Dynamic Thermal Regulation Systems

**Figure 2:**
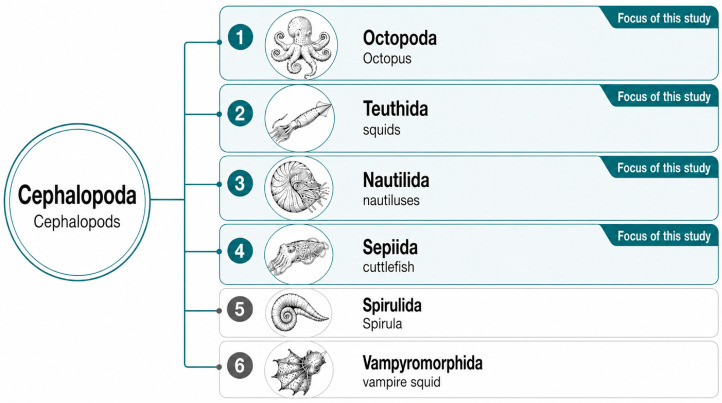
Taxonomic network of Cephalopoda for dynamic thermal camouflage.

Cephalopods, a class within the phylum Mollusca, include octopuses, squids, cuttlefish, and nautiluses. They exhibit a distinctive body plan in which the head and limbs are fused, and the external shell is either reduced or internalized. With their highly developed nervous and visual systems, cephalopods are regarded as one of the most intelligent groups of invertebrates, capable of rapid color change and complex behavioral modulation in response to environmental stimuli [25,26]. As depicted in Figure 2, Cephalopoda encompasses multiple orders characterized by dynamic body architectures and adaptive integumentary systems, forming the biological basis for cephalopod-inspired thermal regulation strategies. In recent studies, squids and cuttlefish have been frequently adopted as model organisms in cephalopod-inspired biomimetic research, while inspiration from octopuses, nautiluses, and even extinct ammonites has also been explored.

#### 2.1.1. Squid-Inspired Optical–Thermal Regulation

Squids belong to the class Cephalopoda within the phylum Mollusca. Their body plan includes eight arms and two longer tentacles that extend from below the head. The arms are lined with suckers used to grasp and envelop prey [27]. The skin is coated with a mucous layer that shields the organism from external stimuli while enabling flexible movement. This layer prevents cellular damage, maintains hydration, and increases surface lubricity [28]. The squid’s body primarily consists of soft tissues composed of collagen fibers, which provide high elasticity and mechanical strength. The skin consists of an epidermal layer and a dermal layer, within which a pigment cell layer is located in the upper region of the dermis. This pigment cell layer is primarily composed of chromatophores and iridophores, while leucophores may also be present depending on the species. These specialized pigment cells enable rapid and dynamic modulation of skin coloration [29]. In squid-inspired thermal management technologies, the pigment cell layer has been one of the most frequently referenced biological features. Squids actively regulate their skin coloration for camouflage through this layer, and numerous biomimetic studies have drawn on this mechanism from an optical perspective, particularly for infrared modulation.

**Figure 3:**
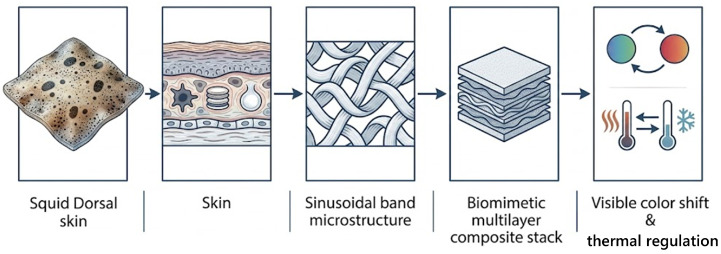
Bio-inspired dynamic thermal regulation system mimicking squid skin.

Bogdanov et al. [16] investigated the iridophores in the dorsal mantle skin of Doryteuthis pealeii and revealed a gradient refractive index distribution within these reflective cells. Using holotomography and confocal microscopy, the researchers confirmed that the internal structure of the iridophores is not a simple multilayered system but instead exhibits a sinusoidal refractive index pattern ranging from 1.35 to 1.42. This gradient enables smooth spectral transitions from transparency to red, orange, and green [16]. Mechanistically, the continuous refractive-index gradient reduces abrupt optical discontinuities between adjacent domains and distributes phase retardation across the cell, thereby enabling gradual wavelength selection rather than sharp, interface-dominated reflection. As depicted in Figure 3, transitioning from squid dorsal skin to a biomimetic multilayer composite stack, the hierarchical microstructure enables visible color shift and infrared thermal regulation. To mimic this architecture, the team fabricated a composite material by embedding Bragg reflector layers of nanocolumnar metal oxides into an elastic polymer substrate. In this fabrication process, the nanocolumnar oxide layers are particularly important because their porous columnar morphology provides a tunable effective refractive index, while the elastic polymer matrix preserves the multilayer spacing and allows reversible deformation-driven optical modulation. Under strains of up to 150% and in environments with solvent refractive indices below 1.56, the material showed tunable wavelength shifts (Δλ≈90nm) and controllable transparency. By adding metallic nanofilm layers to a multilayer structure, the composite achieved multispectral responsiveness, allowing modulation of thermal emission across both visible and infrared regions. These metallic nanofilms function as infrared-active radiative elements within the same process-defined multilayer architecture, linking visible structural coloration with radiative heat exchange for adaptive thermal regulation. Under different stimuli, the surface temperature changed from −18 °C to +4 °C. The composite also maintained its optical and thermal performance at elongation strains up to 790% and after over 25,000 deformation cycles. These results clarify the structural coloration mechanism in squid skin and demonstrate that mimicking its pigment cell layer enables materials capable of adaptive optical and thermal regulation across the visible and infrared spectra. Such chromatophore-inspired designs highlight the strong potential of cephalopod biology for advanced thermal management technologies [16].

#### 2.1.2. Cuttlefish-Inspired Porous Insulating Systems

Cuttlefish belong to the class Cephalopoda within the phylum Mollusca. Their bodies are broad and dorsoventrally flattened, with paired lateral fins running along the mantle. They possess eight arms and two longer tentacles positioned beneath the head, each lined with suction cups used for capturing and securing prey [30]. One of the most distinctive anatomical features is the internal calcareous structure known as the cuttlebone, located dorsally within the mantle. The cuttlebone is made of aragonite and has a micro-porous design consisting of chambers separated by thin septa. By regulating the ratio of gas to liquid inside these chambers, cuttlefish can precisely control their buoyancy. This multilayered lattice structure resists external pressure and allows efficient vertical movement with minimal energy consumption [31]. The skin of the cuttlefish is covered by a thin mucous layer that offers protection from external stimuli while reducing friction and minor mechanical damage. This layer also improves hydrodynamic stability, supporting efficient movement and overall survivability [32].

**Figure 4:**
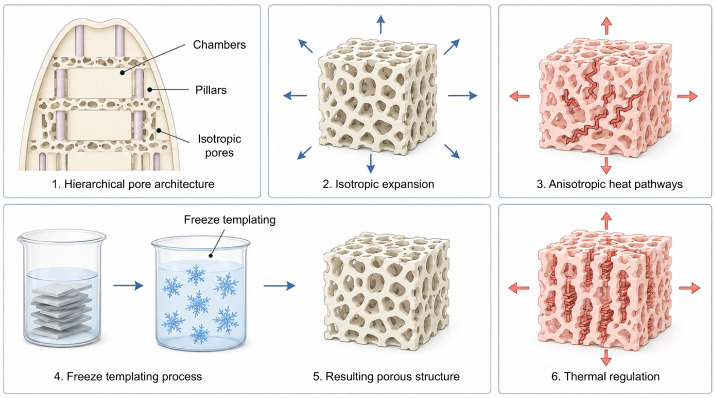
Fabrication process and structural analysis of the bio-inspired porous material.

In bioinspired thermal management materials, the key advantage of the cuttlebone lies in its combination of ultralight architecture and hierarchically porous structure. This unique design suppresses heat transfer in an isotropic manner. The wall–septa/chamber–pillar framework prevents heat from following a straight through-plane or in-plane route, forcing thermal energy to encounter repeated solid–air interfaces and tortuous pore walls in multiple directions. The cuttlebone maintains mechanical strength despite its high porosity, making it one of nature’s most efficient thermal insulators. Recent studies have shown that the chamber–pillar lattice and multiscale porosity of the cuttlebone effectively lengthen heat transport pathways and enhance phonon scattering. As a result, the thermal conductivity decreases uniformly in all directions. Micro-computed tomography and scanning electron microscopy analyses reveal that the pores are arranged in a complex mixture of vertical and horizontal orientations, rather than aligned along a single axis. To replicate this structure, researchers have used isotropic freeze-templating with $Ti_{3}C_{2}T_{x}$ MXene nanosheets to fabricate aerogels. By controlling ice crystal growth during freeze casting, they produced an isotropic porous network resembling the cuttlebone. During freeze-templating, the growing ice crystals operate as sacrificial structural templates that redistribute MXene-based building blocks into wall–septa-like domains; after ice removal, the retained scaffold preserves multidirectional thermal barriers. The resulting aerogels maintained structural integrity even at ultralow densities of 8–15 $\mathrm{mg}\cdot {\mathrm{cm}}^{3}$. As depicted in Figure 4, this natural isotropic architecture was translated into a freeze-templated MXene aerogel with a similarly multidirectional porous network. These aerogels achieved thermal conductivities between 0.021 and 0.025 $W\cdot m^{-1}\cdot K^{-1}$, comparable to that of air. Their excellent insulation arose from multidirectional phonon scattering and the intrinsic surface properties of MXene nanosheets. The insulation mechanism is therefore coupled: high porosity lowers gas/solid heat conduction, nanoscale pores restrict molecular motion through the Knudsen effect, and tortuous MXene-based walls extend the solid conduction path. The materials also showed minimal mechanical degradation after over 10,000 compression cycles and retained their insulation under elevated temperatures without pore deformation [17]. These findings demonstrate that the natural thermal insulation mechanism of the cuttlebone can be effectively translated into MXene-based ultralight aerogels. Such bioinspired materials hold strong potential for applications requiring precise heat-flux control, including battery protection layers and thermal shielding for aerospace systems.

#### 2.1.3. Octopus-Inspired Adaptive Interface Systems

An octopus is a shell-less, soft-bodied marine organism characterized by a compact mantle and eight highly muscular arms bearing rows of suckers. Unlike finned octopods, octopuses lack cirri, paired fins, and an internal shell, and their body support is achieved entirely through muscular and connective tissue architectures rather than rigid skeletal elements [33,34]. Each arm functions as a muscular hydrostat, enabling complex bending, elongation, torsion, and force generation without skeletal support. The suckers generate reversible adhesion through controlled sub-ambient pressure within an enclosed cavity, mediated by coordinated contraction of radial, circular, and meridional muscle fibers [35,36]. This architecture allows strong attachment to irregular and smooth substrates while maintaining high degrees of manipulation and locomotor versatility. The feeding apparatus consists of a chitinous beak and radula enclosed within the buccal mass, reflecting a predatory trophic strategy. Structurally, octopuses represent a highly derived lineage defined by complete reliance on muscular-hydrostatic organization for support, movement, and environmental interaction [34].

**Figure 5:**
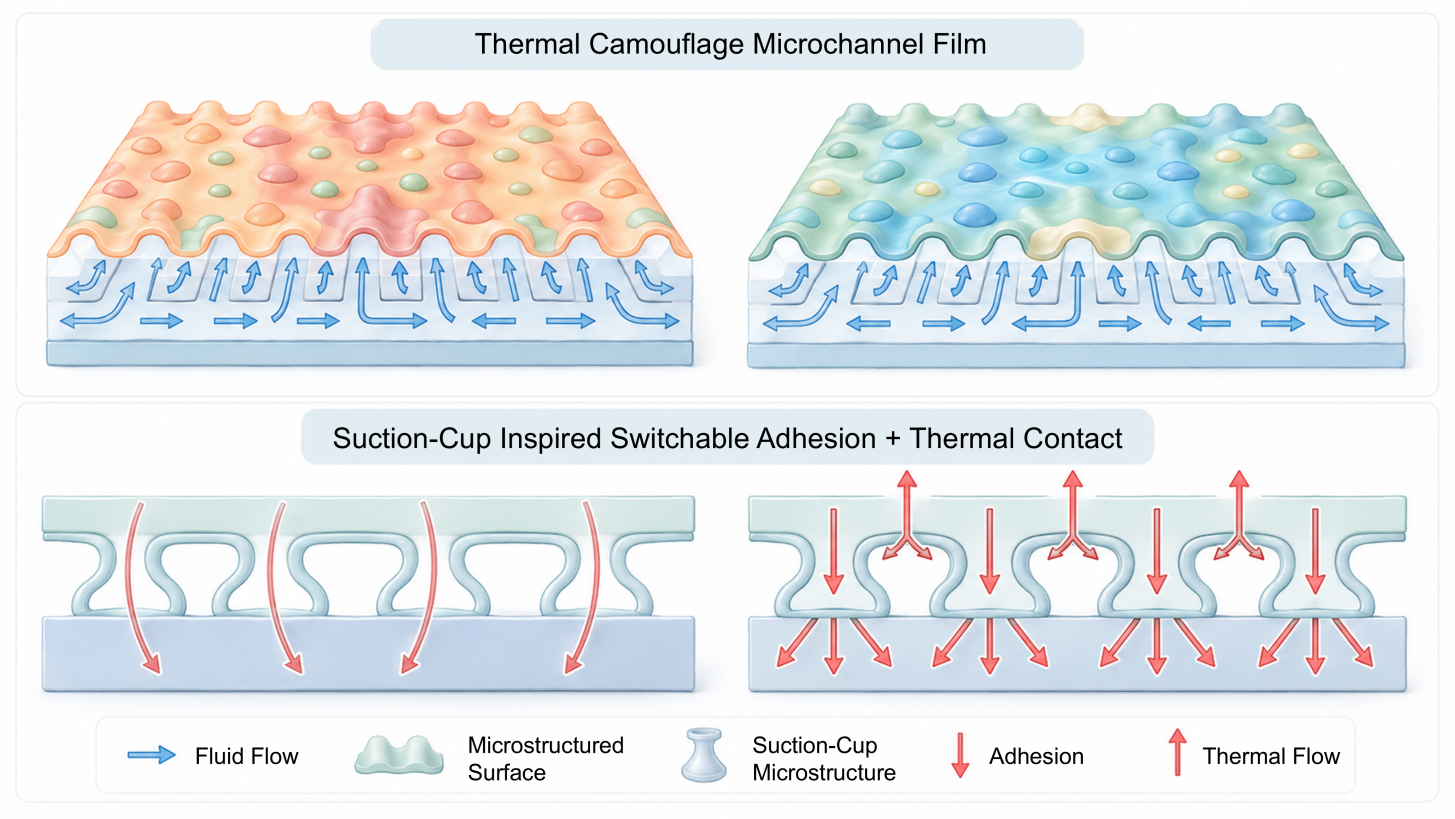
Octopus-inspired adaptive thermal management system.

Unlike squids and cuttlefish that primarily rely on pigment-based coloration, octopuses (Octopus) can rapidly modulate surface morphology and temperature distribution through finely controlled muscular layers and dynamic skin patterning. This dynamic surface modulation provides an engineering cue for controlling apparent thermal signatures because local changes in texture, surface area, and boundary-layer contact can alter radiative emission and convective heat exchange at the skin–environment interface. In one representative study, octopus skin papillae were mimicked to fabricate a micro-patterned thermal camouflage film incorporating microchannel networks. Figure 5 illustrates the octopus-skin–inspired microchannel thermal camouflage film, enabling precise surface temperature control and infrared suppression. Because the channels are integrated beneath the patterned surface, the thermal input can be spatially distributed across the film rather than concentrated at a single heat source, allowing the apparent infrared signal to be regulated through local heat-flux redistribution. Uniform flow distribution within the channels enabled precise surface temperature control within a range of ±8–15 °C. Under the reported experimental conditions, infrared (IR) detection signals were reduced by up to approximately 70%, demonstrating the effectiveness of octopus-inspired micro-patterning as a thermal camouflage design strategy [18]. This reduction in IR detectability can be interpreted as a coupled effect of microchannel-mediated temperature regulation and surface-pattern-induced disruption of a spatially uniform thermal signature. Furthermore, octopus-inspired deformable multicamouflage textiles have been shown to dynamically adjust surface arrangements and thermal diffusion pathways in response to environmental conditions, resulting in approximately 1.5–2 times increases in tunable thermal diffusivity. In addition, switchable adhesion systems inspired by octopus suckers have been reported to reduce thermal contact resistance and enhance heat transfer coefficients by more than twofold compared to conventional interfaces [19]. For thermal interfaces, the suction-cup mechanism is especially important because controllable negative pressure and conformal deformation can reduce interfacial air or water gaps, increase real contact area, and lower thermal boundary resistance. Collectively, these findings indicate that octopus-inspired interfacial architectures can serve as key platforms for stable thermal transport in applications such as attachable thermal sensors, cooling modules, and surface cooling systems for underwater robotic platforms.

#### 2.1.4. Nautilus-Inspired Spiral Flow Systems

Nautilus is the only extant genus within the subclass Nautiloidea and represents the most basal surviving lineage of externally shelled cephalopods. Molecular and karyological evidence indicates that nautiloids retain ancestral genomic traits among living cephalopods, including an unusually long 18S rDNA sequence and the lowest chromosome number reported in the class (2n = 52) [37]. Phylogeographic analyses based on mitochondrial COI and 16S markers further reveal strong geographic structuring across Indo-Pacific populations while indicating limited species-level divergence within historically described taxa [38]. Morphologically, Nautilus is characterized by a planispirally coiled external shell partitioned into gas-filled chambers by nacreous septa and penetrated by a central siphuncle that regulates buoyancy. The shell wall exhibits a hierarchical aragonitic composite architecture consisting of an outer porcellaneous (granular–prismatic) layer and a thick inner nacreous layer composed of polygonal aragonite platelets separated by organic interlayers [39]. This dual-layer structure integrates surface hardness with fracture-resistant toughness, defining Nautilus as a phylogenetically basal yet structurally optimized cephalopod lineage.

**Figure 6:**
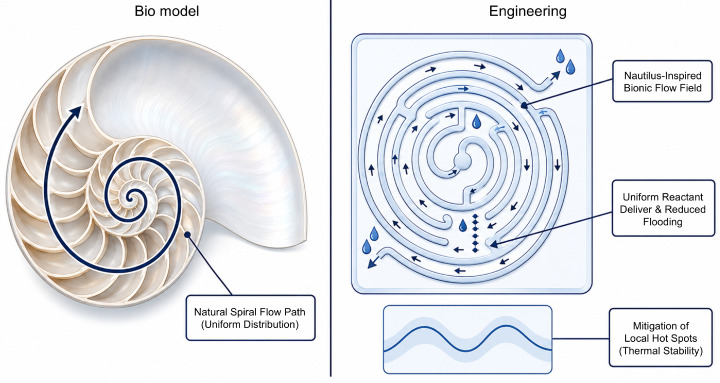
Bio-inspired flow field design based on the Nautilus shell.

The bio-to-engineering translation of the nautilus spiral geometry is schematically presented in Figure 6. Mechanistically, the nautilus-derived spiral geometry provides a gradual radial-to-circumferential transport pathway, allowing fluid, heat, or mass flux to be redistributed over an expanding area rather than being concentrated along a single linear channel. Li et al. [40], In this study, a three-dimensional CFD-based PEMFC architecture incorporating a nautilus-inspired spiral channel was developed to improve flow uniformity. This design effectively suppressed flooding, enhanced reactant gas transport, and increased peak current density by approximately 46.7% and peak power density by 21.5%. The improvement in electrochemical output can therefore be attributed to the coupled regulation of oxygen supply, liquid-water removal, and local current-density uniformity, which reduces mass-transfer losses during high-load operation. The spiral geometry further mitigated localized hot spots and reduced spatial gradients in heat and mass transfer, thereby enhancing the thermal stability of the fuel cell [40]. Beyond fuel cells, nautilus-inspired multilayer logarithmic spiral walls have also been applied to phase change material (PCM) thermal energy storage systems, where they shortened melting and solidification times while promoting uniform temperature distribution. Likewise, spiral volute geometries inspired by the nautilus have demonstrated reduced pressure loss in blower and fan designs [41]. For blower and fan systems, the gradually expanding spiral volute guides rotating flow toward the outlet while suppressing adverse pressure gradients, recirculation near the volute tongue, and vortex-induced energy loss. Overall, although nautilus-derived geometries do not directly modify intrinsic thermal conductivity, they are highly effective as system-level thermal management strategies by enhancing flow distribution, homogenization, and mixing efficiency.

### 2.2. Gastropod-Inspired Shell-Based Thermal Architectures

**Figure 7:**
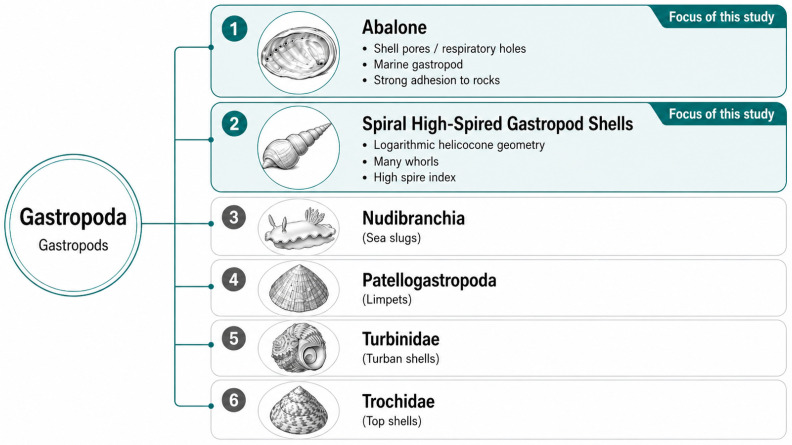
Classification network of Gastropoda for robust thermal protection.

As classified in Figure 7,Gastropods include snails and slugs inhabiting marine, freshwater, and terrestrial environments worldwide [42]. Their body plan generally includes a muscular ventral foot, head region, and visceral mass enclosed by a single external shell, although shell reduction or loss has evolved in multiple lineages [43]. Torsion produces the asymmetrical adult body organization of gastropods [44]. The engineering relevance of Gastropoda lies in biomineralized shell geometries ranging from perforated abalone forms to helicoconical, high-spired architectures. Abalone-like nacre supports mechanically robust layered composite design [20] and directional thermal-conductive pathways in BNNS/epoxy layered nanocomposites [21], whereas high-spired shells support vibration attenuation, stress redistribution, and thermo-mechanical protection [22].

#### 2.2.1. Abalone-Inspired Directional Heat Conduction Systems

Abalone(Haliotis) are marine gastropods with a dorsoventrally flattened, perforated shell [45] and ecological and economic relevance in shallow rocky coasts [46]. Their shell is a hierarchical biocomposite consisting of an outer calcitic prismatic layer and an inner nacreous layer of aragonitic tablets separated by thin organic interlayers [47]. Nacre forms through phase regulation and mineral-bridge-connected columnar stacking, producing high fracture resistance [48]. Although shell morphology and molecular lineage information remain relevant to systematics [45,49], the thermal-management relevance of abalone nacre arises from its layered mineral–organic architecture, which supports mechanically robust and multifunctional nacre-inspired structures [20] and anisotropic thermal-conductive pathways in nacre-inspired BNNS/epoxy layered nanocomposites [21].

**Figure 8:**
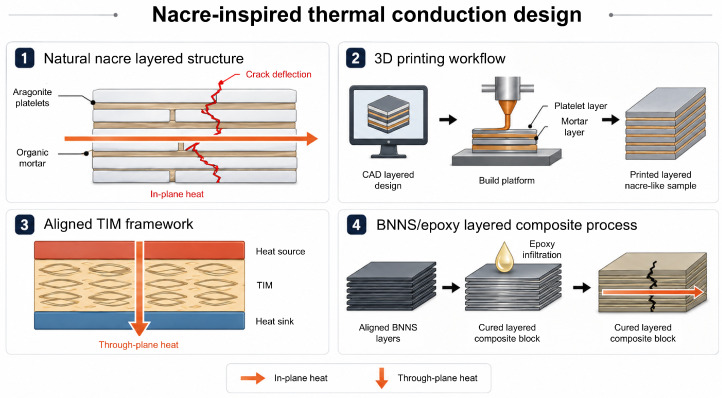
Nacre-inspired thermal conduction design.

The nacreous layer of the Abalone shell consists of alternating stacks of aragonite platelets and organic mortar layers. This hierarchical “brick-and-mortar” architecture provides multiple mechanical toughening mechanisms, including crack deflection, crack arrest, and interlayer sliding. Recent studies published within the past five years that emulate these structural features have reported composite materials that achieve enhanced thermal conductivity, mechanical durability, and fracture toughness simultaneously. The bio-to-engineering translation of nacre’s brick-and-mortar architecture into directional thermal conduction frameworks is schematically summarized in Figure 8. Yang et al. [20] directly replicated the hierarchical architecture of abalone nacre using additive manufacturing. In this work, artificial nacre was fabricated by alternately stacking platelet-like layers (serving as aragonite analogues) and compliant organic mortar layers. Experimental results confirmed pronounced crack deflection and increased energy absorption within the printed structures. In selected specimens, nanoparticles were incorporated to impart additional electrical and thermal functionalities, demonstrating that the multifunctional synergy inherent to natural nacre can be reproduced through process-driven fabrication strategies [20]. Furthermore, Wang et al. [21] reported a layered hybrid composite that mimics the platelet–mortar architecture of abalone nacre using BNNS and epoxy. Although BNNS exhibits intrinsically high thermal conductivity, random dispersion hinders the formation of continuous heat-transfer pathways. To address this limitation, the authors adopted a nacre-inspired hierarchical stacking strategy, aligning BNNS into layered architectures that establish uninterrupted thermal conduction channels. This approach yielded a pronounced increase in in-plane thermal conductivity, together with high mechanical strength and superior fracture toughness, indicating effective reproduction of nacre-like crack deflection and crack-arrest mechanisms within the composite [21]. Collectively, these studies share a common strategy of exploiting nacre-inspired directional control of thermal conduction pathways while simultaneously enhancing structural stability, strength, and toughness, underscoring the effectiveness of abalone nacre as a bioinspired blueprint for multifunctional thermal management materials.

#### 2.2.2. Spiral High-Spired Gastropod Shells as Vibro–Thermo–Mechanical Archetypes

Spiral high-spired shells are helicoconical architectures formed by logarithmic coiling and axial elongation [50], with elevated spire geometry reflecting constraints related to gravitational stability and locomotion [51,52]. They contain crossed-lamellar microarchitectures with first-, second-, and third-order lamellae, where aragonitic elements of approximately 100–150 nm are alternately oriented [22,53]. In Turritella, helicoconical geometry and lamellar hierarchy jointly influence global modal characteristics and vibration attenuation [22]. This geometry–lamella coupling can serve as a structural template for stress redistribution, thermal transport pathways, and stability under coupled thermal and mechanical loading [22].

**Figure 9:**
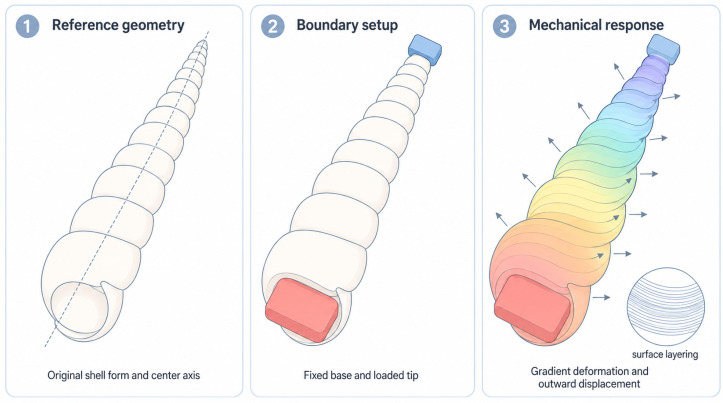
Thermal dissipation mechanism inspired by the helical shell structure.

Compared with oyster shells, whose nacreous layers have been extensively explored as biomimetic models, thermal management technologies inspired by other marine gastropods such as turret shells, limpets, and nudibranchs have received relatively limited attention. However, recent studies indicate that marine gastropods possessing spiral outer shells, including Turritella and conch species, may serve as effective biological templates for biomimetic design. Turritella terebra and Turritellinella tricarinata exhibit elongated spiral morphologies with finely layered lamellar architectures, which provide resistance against repeated mechanical impacts and predation in marine environments. The spiral geometry increases the density of vibrational modes in the high-frequency regime compared with simple conical shells. As a result, localized concentration of vibrational energy is effectively suppressed, as illustrated in Figure 9. A 2023 study entitled “Multiscale static and dynamic mechanical study of Turritella shells” employed μ-CT–based three-dimensional morphological analysis together with nanoindentation (AFM modulus mapping). This approach enabled simultaneous characterization of the microstructural and macrostructural features of the spiral shells. Cross-sectional analysis revealed that the thickness of third-order lamellae was uniformly distributed at approximately 120–150 nm [22]. Based on these structural parameters, finite element models were constructed to predict natural frequencies and mode shapes. The simulated response showed high agreement with experimentally measured resonance spectra obtained via resonant ultrasound spectroscopy. Through inverse estimation, the effective elastic modulus of the shell was determined to be approximately 53 GPa, and the loss factor was estimated to range from approximately $5\times 10^{-3}$ to $1\times 10^{-2}$. These results suggest that the shell does not behave as a simple viscoelastic material. Instead, spiral morphological factors such as spiral pitch and curvature gradients directly contribute to the distribution of dynamic loads [22]. Similar trends were observed in composite materials designed to mimic the spiral shell architecture. When spiral internal ribs and lamella-like micro-patterns were implemented within composite structures, the number of resonance peaks increased in the high-frequency regime (above 100 kHz). At the same time, resonance amplitudes decreased by approximately 35–50% compared with geometrically simple conical composites of identical mass and external dimensions. During thermal shock testing, spiral-structured composites also exhibited a delay in crack initiation of approximately 28%, indicating enhanced thermo-mechanical stability. These observations suggest that spiral architectures promote more distributed stress flow and potentially expanded thermal transport pathways, contributing to improved protective performance under coupled thermal and mechanical loading [22]. The structural complexity of spiral-wound heat exchanger designs, compared with conventional shell-and-tube configurations, has also been highlighted in the work of Q. Gong et al. [54]. During fabrication, spiral piping requires precise control of the winding radius. Maintaining uniform interlayer spacing and ensuring reliable fixation are considerably more challenging than in straight-tube systems. In addition, the continuously varying spiral curvature imposes stricter requirements on manufacturing equipment and increases the complexity of welding and joint design [54]. Although spiral architectures are more difficult to manufacture than conventional linear designs, continued advances in spiral-based fabrication technologies could enable efficient manufacturing processes. If such processes are realized, spiral structures may achieve higher heat transfer efficiency within the same volume, offering strong potential competitiveness for compact, high-performance heat exchanger designs.

### 2.3. Bivalve-Inspired Lamellar Thermal Control Systems

**Figure 10:**
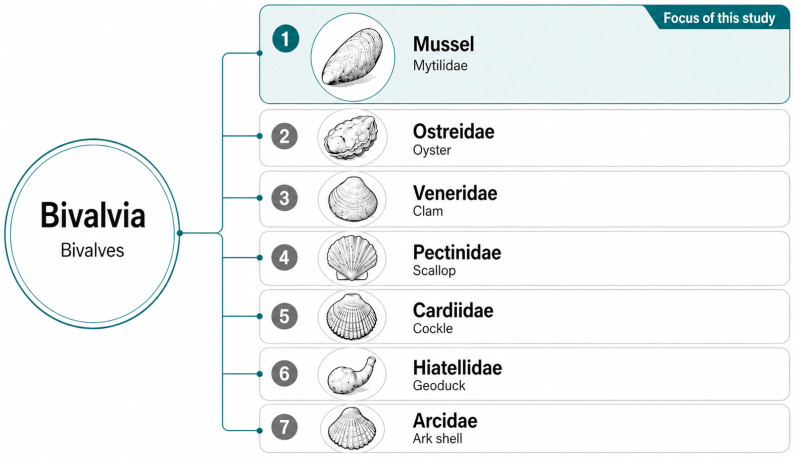
Systematic network of Bivalvia for passive thermal regulation.

Bivalvia is a class within the phylum Mollusca characterized by a body laterally compressed and enclosed by two calcareous shell valves that are articulated dorsally by a hinge and ligament system. The opening and closing of the paired valves are controlled by anterior and posterior adductor muscles. This bivalved shell architecture constitutes the most direct and diagnostically distinctive morphological feature that clearly differentiates bivalves from other molluscan classes [55]. The taxonomic framework and representative lineages of Bivalvia are schematically presented in Figure 10. Unlike most other mollusks, bivalves have completely lost the radula, and instead exhibit highly specialized gills (ctenidia) that function not only in respiration but also as the primary apparatus for filter feeding. In the majority of species, the gills capture suspended particles from the surrounding water through coordinated ciliary motion and mucus secretion. This functional transformation of the gills represents a central biological mechanism in bivalve evolution and serves as a major criterion underlying phylogenetic differentiation between Protobranchia and Autobranchia [56]. The soft body is enclosed by the mantle, which plays a central role in shell formation, regulation of shell microstructure, and, in some taxa, responsive modification to environmental stimuli. The microstructural organization of the shell—such as prismatic and nacreous layers—together with hinge dentition patterns, is reliably preserved in the fossil record. Consequently, bivalves provide one of the most continuous and informative fossil archives among metazoans, extending back to the early Cambrian [55]. Ecologically, bivalves exhibit a wide range of life habits, including infaunal burrowing, epifaunal attachment, boring, and free-swimming lifestyles. These ecological strategies are accompanied by functional differentiation of the foot, mantle margins, ligament systems, and gill morphologies. Such tightly coupled relationships between morphology and function position bivalves as a particularly valuable model for investigating biological structural design principles that optimize environmental adaptation and material–energy exchange.

#### Mussel-Inspired Wet-Stable Interfacial Thermal Systems

Mussels are epibenthic, byssally attached pteriomorph bivalves distinguished by a posteriorly expanded, inequilateral shell and lifelong secretion of collagen-based attachment threads [57,58]. The shell typically consists of an outer calcitic prismatic layer and inner aragonitic nacreous layers, integrating stiffness, fracture resistance, and growth plasticity across environmental gradients [58]. Mechanical stability, however, is primarily governed by the byssus—an extracorporeal tension-bearing system composed of hybrid block-copolymer collagens (preCols) that combine a central collagen domain with elastin-like, silk-like, or glycine-rich motifs [59,60]. The axial gradient in preCol distribution generates spatial modulation of stiffness and extensibility along each thread, producing a reversible, shock-absorbing attachment mechanism distinct from vertebrate tendon systems [60]. Through the integration of hierarchical shell architecture and mechanically graded byssal threads, mussels achieve robust substrate anchorage, hydrodynamic resilience, and ecological dominance in intertidal and subtidal environments [58].

**Figure 11:**
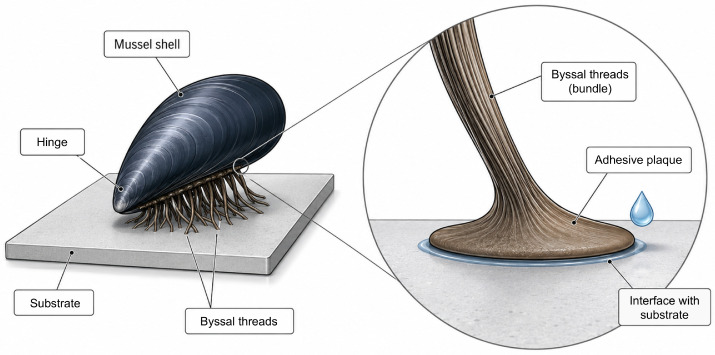
Adhesion mechanism of mussel byssal threads.

Zhang et al. [23], catechol groups were introduced into a PDMS matrix by copolymerizing dopamine methacrylamide (DMA), thereby emulating the catechol–metal coordination chemistry of MAPs. The fabrication schematic of this example is presented in Figure 11. This design enabled stable dispersion of high-surface-tension liquid metal EGaIn into nano- and microscale droplets. Mechanistically, catechol units coordinate with the oxide skin of EGaIn droplets, allowing the PDMS matrix to encapsulate and separate liquid-metal domains while forming shorter and more stable heat-transfer pathways. The resulting composite achieved a thermal conductivity of 6.9 $W\cdot m^{-1}\cdot K^{-1}$ and an elastic modulus of 75.8 kPa, while maintaining structural and functional stability under wet conditions. These results demonstrate that mussel-inspired adhesion mechanisms can be directly translated into underwater thermal management and flexible thermal interface materials [23]. Marine mussels possess multilayered protective coatings attributed to a laminated brick-and-mortar architecture that resists pressure, oxygen, and moisture ingress. Inspired by this structure, researchers developed a directionally aligned multilayer coating using cellulose nanofibers (TOCN) as the mortar and mica–$TiO_{2}$ platelets as the brick phase. The coating achieved a solar reflectance (R_solar) of 0.90 and long-wave infrared emissivity (ϵ_LWIR) of 0.94, enabling subambient daytime cooling of −5.3 °C. From a thermal-process perspective, the aligned platelet layers function as a coupled optical–thermal barrier, where solar heat input is suppressed by backscattering while absorbed heat is released through long-wave infrared emission. The layered mica–$TiO_{2}$ architecture also improved flame retardancy, maintaining 45% power output after 20 s of direct flame exposure. Additionally, TOCN-enabled electrostatic charge accumulation supported triboelectric energy harvesting, while the interlocked network structure provided mechanical recyclability with substantial retention of cooling and energy-harvesting performance [23]. A 2024 review in *ACS Biomaterials Science and Engineering* systematically analyzed the structural and chemical characteristics of MAPs and polydopamine (PDA) in electrospun nanofiber-based materials. DOPA-lysine motifs impart interfacial stability under humid, saline, and underwater conditions through metal coordination, π–π interactions, hydrogen bonding, Michael addition, and Schiff base reactions. PDA consolidates this catechol-based chemistry into a single polymeric system, offering potential for photothermal regulation, stabilization of thermal conduction pathways at polymer–inorganic interfaces, and underwater thermal interface design [24]. At polymer–inorganic interfaces, these catechol/PDA layers can act as molecular coupling bridges that improve wetting and contact continuity, thereby reducing interfacial thermal resistance and stabilizing heat flow under hydrated conditions. Despite targeting different applications, these studies converge on three mussel-inspired principles: wet-stable interfacial adhesion, catechol-based multivalent interactions, and hierarchical protective architectures. Reported improvements include enhanced thermal conductivity via liquid metal dispersion, radiative heat control and flame retardancy through layered designs, and reduced interfacial thermal resistance enabled by stable adhesion. Collectively, these advances span thermal conduction, heat dissipation, and underwater heat transfer stabilization, outlining clear design directions for biomimetic thermal management technologies.

## 3. Thermal Management Strategies Inspired by Porifera

**Figure 12:**
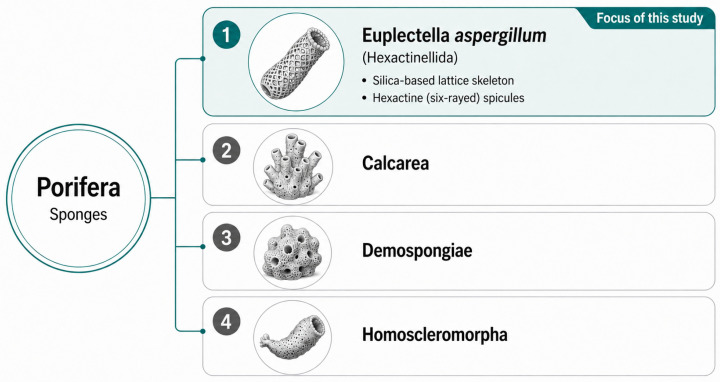
Structural hierarchy of Hexactinellida for high-efficiency thermal insulation.

Porifera is a phylum of early-diverging metazoans characterized by a multicellular body organization lacking true tissues and organs, in which physiological integration is achieved through coordinated cellular functions. The sponge body is penetrated by a well-developed aquiferous system consisting of ostia, internal canals, and oscula, enabling continuous water flow for suspension feeding, respiration, and waste removal [61]. The body wall is organized into an outer pinacoderm, an inner choanoderm lining flagellated chambers, and an intervening mesohyl containing diverse cell types and skeletal components. Choanocytes act as the primary functional units, generating water currents and capturing particulate matter through collar–flagellum complexes [62]. Structural support is provided by a skeleton composed of mineral spicules and/or organic fibers embedded within the mesohyl. The morphology, symmetry, and arrangement of spicules are genetically controlled and constitute the principal criteria for sponge taxonomy and higher-level classification [63]. Based on differences in skeletal composition, tissue organization, and developmental traits, extant sponges are classified into four major classes—Demospongiae, Calcarea, Hexactinellida, and Homoscleromorpha. Molecular and morphological evidence supports Porifera as a distinct evolutionary lineage while revealing deep divergence among its constituent classes, particularly the syncytial organization unique to Hexactinellida [64]. The major poriferan classes are summarized in Figure 12.

### Glass Sponge–Inspired Structure-Induced Flow Organization Systems

Hexactinellida is a class of exclusively marine sponges within the phylum Porifera, defined by a skeletal system composed entirely of siliceous spicules organized according to a fundamentally triaxial structural principle. The hexactine represents the central morphological archetype of this system, from which other megasclere forms are derived through systematic reduction of primary rays, while retaining an internal axial cross characteristic of triaxonic symmetry [65,66]. The skeletal framework lacks both calcareous minerals and organic spongin and may consist of discrete spicules or secondarily fused elements forming rigid dictyonal architectures. Such fusion enhances mechanical rigidity but is not universal across the class and therefore does not constitute a defining diagnostic feature [67]. At the tissue level, hexactinellids exhibit a distinctive syncytial organization in which large regions of the body, including dermal and atrial membranes, form continuous multinucleate cytoplasmic domains connected by specialized porous junctions. This biological architecture represents a fundamental departure from the cellular organization observed in other sponge classes [65]. Hexactinellida are exclusively marine and predominantly inhabit deep-sea environments. Their functional morphology and spicule systems are best understood within a phylogenetic framework emphasizing spicular architecture rather than external appearance or degree of spicule fusion, an approach long advocated in classical systematic treatments [66,67]. Modern taxonomic treatments and descriptive studies of extant hexactinellids continue to employ this structural framework, applying it consistently across both classical taxa and newly described species, thereby reinforcing the coherence of Hexactinellida as a distinct evolutionary lineage [68].

**Figure 13:**
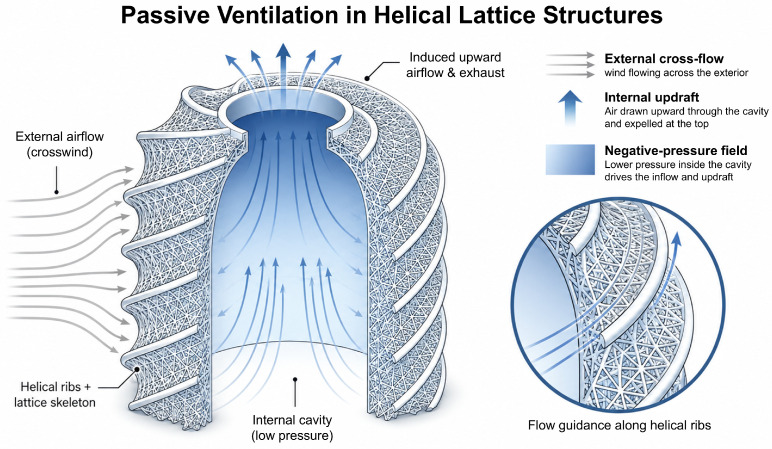
Mechanism of passive ventilation in sponge-inspired helical lattice structures.

Figure 13 illustrates the helical lattice skeleton of Euplectella aspergillum and the resulting passive flow-organization mechanism, characterized by external cross-flow guidance, internal negative pressure formation, and induced upward ventilation. Falcucci et al. [69] reported that ultra-high-resolution CFD simulations based on a precise reconstruction of the real skeletal geometry of Euplectella aspergillum revealed a flow-organization mechanism. In this mechanism, external flow is guided along the spiral ridges of the outer skeleton, generating a negative pressure gradient inside the central cavity and thereby inducing spontaneous upward flow. In terms of thermal-process design, this geometry can be interpreted as converting lateral cross-flow into axial through-cavity advection, which renews the fluid near the internal skeleton and provides a structural basis for convection-assisted heat and mass exchange without active pumping [69]. Across a wide Reynolds number range of 5–5000, the cavity-averaged vertical velocity ⟨$u_{z}$⟩ remained consistently positive. In particular, at Re ≈ 100, the ratio of outflow through the osculum to the inflow reached a maximum of approximately 37%. This behavior was interpreted as a functional adaptation to deep-sea environments, wherein weaker external flows paradoxically lead to enhanced flow-collection efficiency due to the skeletal architecture. Such flow organization can be further understood as a structural feature that passively creates favorable conditions for heat and mass exchange by increasing residence time within the central cavity. The helical ridges and porous windows can therefore be viewed as a passive flow-organizing interface, coupling pressure-gradient formation with residence-time control rather than relying on externally supplied pumping energy [69]. These biological principles have been extended to engineering materials. Luo et al. [70] mimicked the multilevel skeletal architecture of deep-sea glass sponges to fabricate a graphene aerogel with continuously connected cellular structures across nano-, micro-, and macro-scales. The six-level hierarchical network dispersed localized deformation while preserving global resilience, retaining 99.9% of its original height after 20,000 compression cycles at 90% strain. Beyond mechanical durability, the porous architecture maintained continuous internal pathways and an open cellular framework, which may provide an architectural basis for air-mediated and surface-mediated heat transfer. From a thermal-design perspective, the connected carbon-wall framework can provide potential solid-conduction routes, while the retained pore network preserves space for gas-mediated transport; thus, the hierarchical skeleton may support coupled conduction–convection pathways under mechanical deformation. Preservation of pore integrity under extreme deformation further sustained conductive and convective heat-transfer pathways during repeated mechanical compression cycling [70]. In summary, sponge-inspired biomimetic research integrates flow collection, flow-path organization, and residence-time control into passive thermal-management strategies. These mechanisms promote convection-driven heat transfer and autonomously regulate internal heat and mass exchange without external energy input. Accordingly, sponge-derived structure–flow interactions provide effective design principles for low-energy heat exchangers, passive cooling systems, and porous thermal-diffusion matrices.

## 4. Thermal Management Strategies Inspired by Cnidaria

**Figure 14:**
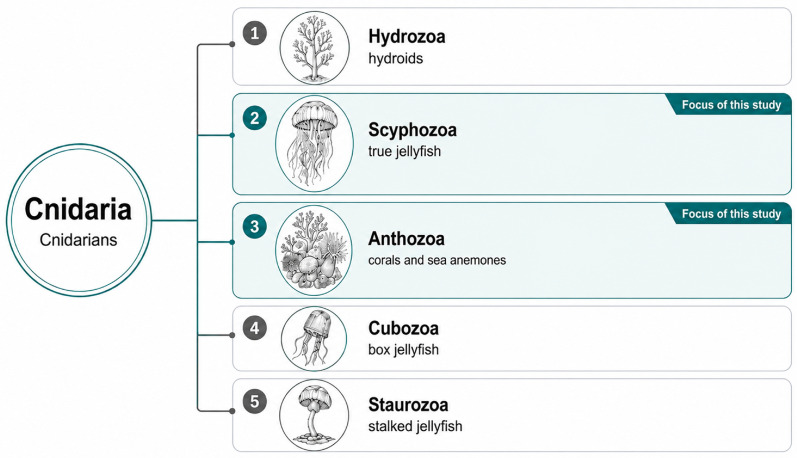
Phylogenetic network of Cnidaria for microfluidic heat exchange.

Cnidaria is a phylum of primarily aquatic metazoans characterized by the presence of cnidae, specialized intracellular organelles capable of rapid discharge, which represent the most diagnostic and unifying feature of the group. Cnidarians exhibit a diploblastic body organization composed of an ectoderm and endoderm separated by a gelatinous mesoglea, and lack true organs despite often exhibiting considerable morphological and functional complexity [71]. Members of Cnidaria typically display a life cycle involving one or both of two distinct body forms: a sessile polyp and a free-swimming medusa, although the medusa stage is entirely absent in Anthozoa. This polyp–medusa dichotomy has been central to interpretations of cnidarian evolution, life-history strategies, and morphological diversification [72,73]. Figure 14 outlines the structural and phylogenetic framework of Cnidaria, showing divergence across major lineages. From a phylogenetic perspective, Cnidaria is broadly divided into two major clades: Anthozoa, comprising exclusively polypoid forms such as corals and sea anemones, and Medusozoa, which includes Hydrozoa, Scyphozoa, Cubozoa, and Staurozoa and is generally characterized by the presence of a medusa stage [72,73]. While nuclear and phylogenomic datasets consistently support the monophyly of these major cnidarian lineages, mitochondrial analyses have revealed conflicting signals, highlighting the deep evolutionary history and complex character evolution within the phylum [73,74]. Beyond their phylogenetic significance, cnidarians exhibit a wide range of functional morphologies, including hydrostatic skeletons, modular body architectures, and diverse extracellular matrices, which together underpin their ecological success and provide rich inspiration for biomimetic material and structural design [75].

### 4.1. Coral-Inspired Multiscale Porous Photothermal Systems

Reef-building corals are colonial anthozoans belonging to the order Scleractinia, characterized by the secretion of an extracellular aragonitic calcium carbonate skeleton that serves as a rigid structural framework for both individual polyps and reef construction [76]. At the macroscopic scale, the skeleton is organized into discrete corallites connected by a common coenosteum, forming an integrated colonial architecture [77]. At the microscopic level, scleractinian skeletons are composed of aragonite fibers radiating from centers of calcification, exhibiting species-specific and taxonomy-linked microstructural arrangements [78]. These fibrous units are not purely mineral precipitates but are intimately associated with an organic matrix distributed at the nano- to microscale, indicating biologically regulated crystallization [78]. Thermogravimetric and infrared analyses further demonstrate that coral skeletons contain up to ∼2–3% organic compounds and structurally associated water, confirming that the coral skeleton is a composite biomineral rather than an abiotic carbonate deposit [79]. Skeletal deposition occurs at the interface between calicoblastic epithelial cells and the extracellular calcifying medium, where ion transport, organic matrix secretion, and environmental factors collectively regulate aragonite precipitation [76]. Accordingly, corals can be defined as biologically controlled aragonitic biomineralizing anthozoans whose skeletal growth integrates genetic regulation, organic matrix mediation, and environmental modulation.

**Figure 15:**
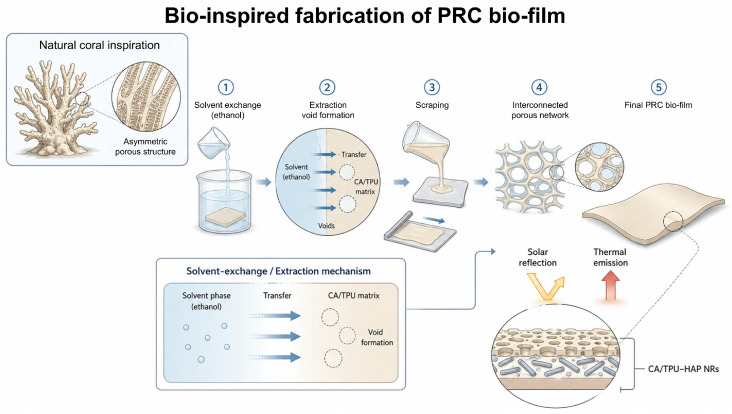
Fabrication process of the coral-inspired passive radiative cooling (PRC) film.

The continuously porous, multiscale architecture of stony coral skeletons enhances photothermal conversion by repeatedly scattering and trapping incident solar radiation within the internal network. In this process, the interconnected pore walls function as distributed heat-generation sites, because multiple internal reflections increase photon–matter interactions before the incident radiation escapes the coating. As shown in Figure 15, the coral-inspired multiscale pore architecture is translated through solvent exchange and extraction into a photothermal coating exhibiting enhanced solar absorption and thermal emission. Guo et al. [80] developed a solar-absorbing coating inspired by this hierarchical porosity and evaluated its long-term thermal and optical stability in concentrating solar thermal (CST) receivers. Replacing conventional alumina ($Al_{2}O_{3}$) binders used in Pyromark-type coatings with rutile-phase $TiO_{2}$ effectively suppressed cation diffusion and thermally induced interfacial delamination at elevated temperatures [80]. Consequently, the thermal process is not limited to light absorption; it also depends on maintaining a stable interfacial pathway for heat transfer by preventing diffusion-driven weakening of the coating–substrate junction. The $TiO_{2}$-based coral-inspired coating maintained an average solar absorptance of 97.39 ± 0.20% after more than 3000 h at 800 °C, with no structural collapse or optical degradation during repeated thermal cycling. In contrast, alumina-based coatings exhibited significant absorptance loss and localized delamination under identical conditions. This stability was attributed to the low cation mobility and oxygen-vacancy-related defect structure of $TiO_{2}$, which blocks high-temperature diffusion pathways. Collectively, these results demonstrate that coral-inspired architectures provide both high initial solar absorption and long-term thermal–optical reliability in high-flux solar thermal environments through coupled structural and materials-level design [80]. In contrast to the aforementioned approaches targeting enhanced solar absorption and thermal stability under high-temperature solar-thermal systems, a distinct case was reported by Cai et al. In this study, coral-inspired architectures possessing calcium-rich porous skeletons were biomimetically replicated to pursue a fundamentally different thermal management objective. Specifically, biomimicry was employed to realize a passive radiative cooling strategy operating in the low-temperature regime. The hierarchically interconnected pore network spanning micro- to macro-scales effectively scattered and reflected incident solar radiation, while simultaneously enhancing thermal emissivity within the atmospheric window (8–13 μm), thereby enabling net heat dissipation without external energy input [81]. For radiative cooling, the heat-flow direction is therefore reversed: solar heat gain is minimized at the surface, while the material’s thermal energy is preferentially released outward as mid-infrared radiation through the emissive porous network. The multiscale pore architecture characteristic of stony coral skeletons facilitates broadband optical responses across a wide spectral range, in contrast to conventional single-scale porous designs. This hierarchical structural configuration enables the simultaneous fulfillment of two inherently different thermal management requirements—solar reflection and infrared emission—within a unified framework. Furthermore, by integrating an elastomeric polymer matrix with inorganic emissive reinforcements, the biomimetic system achieved mechanical compliance and long-term durability without compromising optical performance, even when applied to complex geometries such as building envelopes or vehicle surfaces [81]. Collectively, these findings suggest that stony coral-inspired architectures are not restricted to a single thermal function but instead constitute a versatile thermal management design platform capable of structurally enabling continuous thermal demands ranging from high-temperature solar absorption to low-temperature radiative cooling. Optically, the asymmetrical pore size distribution optimizes Mie scattering across the solar spectrum (0.3–2.5 μm), maximizing backscattering. Concurrently, intrinsic molecular resonances of HAP NRs and polymer functional groups overlap with the 8–13 μm atmospheric window, ensuring highly efficient non-radiative heat dissipation. A critical comparison shows that while metallic/oxide coral structures (Guo et al.) utilize deep cavities for photothermal conversion at extreme temperatures (>800 °C) [80], polymeric asymmetrical networks (Cai et al.) exploit localized diffraction for sub-ambient cooling [81], demonstrating the versatility of the coral motif in manipulating electromagnetic waves for opposite thermal objectives.

### 4.2. Jellyfish-Inspired Hollow Evaporative Thermal Management Systems

Jellyfish are pelagic medusa-stage representatives of the cnidarian clade Medusozoa, a monophyletic lineage that is reciprocally sister to Anthozoa within the phylum Cnidaria [71,75]. Cnidarians are diagnostically defined by the presence of cnidae—intracellular organelle-like capsules that include nematocysts—and a diploblastic body organization consisting of ectoderm and endoderm separated by mesoglea [71,75]. Within Cnidaria, Medusozoa comprises Hydrozoa, Scyphozoa, Cubozoa, and Staurozoa [71,82]. The medusa stage—typically free-swimming and bell-shaped—constitutes the characteristic sexual phase of most medusozoans and is regarded as a derived feature within Cnidaria [82,83]. Medusae generally exhibit radial or biradial symmetry, a gelatinous mesoglea-dominated umbrella, marginal tentacles bearing cnidocytes, and a centralized manubrium associated with the gastrovascular system [84]. Unlike Anthozoa, which are exclusively polypoid, medusozoans typically display a biphasic life cycle including planula larva, sessile polyp, and pelagic medusa, although stage reduction or loss has occurred in several hydrozoan lineages [82,83]. Phylogenomic evidence strongly supports the monophyly of Medusozoa and clarifies deep divergences among its constituent classes [71]. Functionally, jellyfish combine a diploblastic epithelial architecture with well-developed muscle systems and diffuse nerve nets; some taxa possess complex sensory organs such as rhopalia or statocysts [84]. Despite their structural simplicity relative to bilaterians, medusae retain remarkable regenerative capacity, including organ restoration and partial body reconstitution [84]. Accordingly, jellyfish can be rigorously defined as the medusa-stage organisms of Medusozoa characterized by cnidae-bearing diploblastic tissues, mesoglea-supported umbrella morphology, and a life cycle incorporating a pelagic sexual phase within the cnidarian evolutionary framework [71,75].

**Figure 16:**
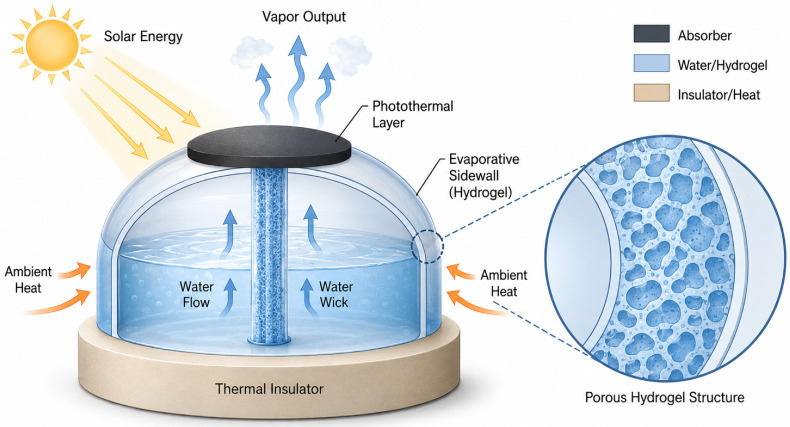
Jellyfish-inspired solar steam generation system.

Jellyfish are often regarded as archetypal ecologically adaptive organisms whose highly hydrated gelatinous tissues and umbrella-shaped hollow bodies maximize heat and mass exchange with the surrounding aquatic environment. This morphology allows them to accommodate environmental fluctuations with minimal active metabolic regulation, as their hollow, water-rich structures naturally equilibrate with external thermal conditions. These characteristics have recently been reinterpreted as sources of bio-inspired design principles for solar-driven evaporative thermal management structures. Xue et al. [85] drew morphological inspiration from the umbrella-shaped hollow geometry of jellyfish to design a selectively three-dimensional hollow evaporator placed in the upper region of the system. As shown in Figure 16, the jellyfish-inspired hollow geometry enables simultaneous solar evaporation and ambient heat harvesting. In this configuration, photothermal conversion at the top surface induces interfacial evaporation, while evaporative cooling simultaneously develops along the non-illuminated sidewalls. As a result, the sidewall surface temperature drops below the ambient temperature, which reverses the direction of radiative and convective heat transfer across the sidewalls and enables the evaporator to passively absorb additional thermal energy from the surrounding environment. Mechanistically, this process can be understood as a coupling between interfacial photothermal heating and lateral evaporative cooling: water continuously supplied by capillary transport evaporates from the sidewalls, carries away latent heat, and thereby establishes an inward radiative and convective heat flux from the surroundings [85]. Infrared thermographic analysis showed that the sidewall temperature of the evaporator was maintained at approximately 3.4 °C below the ambient temperature. Complementary energy balance calculations quantitatively confirmed that, in addition to the incident solar input, a substantial fraction of the evaporation enthalpy was supplied by environmental heat influx. Owing to this environment-assisted heat-absorption mechanism, the system achieved an apparent evaporation efficiency of 108.9%, exceeding the conventional theoretical limit defined solely by the solar energy input. This outcome can be interpreted as an engineering realization of the passive ecological strategy employed by jellyfish, in which hollow, water-rich body architectures minimize net energy dissipation by remaining in near-thermal equilibrium with the surrounding environment [85]. Also, jellyfish-inspired architectures have been translated into both convective heat sinks and interfacial evaporators [86,87]. In microchannel heat sinks, bell-shaped fins induce vortex-enhanced mixing, improving the thermal performance factor (TPF) by up to ≈20.06% and ≈12.93% compared to square fins. In this case, the bell-shaped fin geometry induces flow separation and cavity recirculation, generating local vortices that enhance fluid mixing and convective heat transfer between the coolant and fin surfaces [87]. In hydrogel evaporators, a jellyfish-head core–shell structure stabilizes intermediate water (IW), increasing the IW/FW ratio to 1.60 and reducing the equivalent evaporation enthalpy to ≈$1450\;J\cdot g^{-1}$, enabling $1.90\;\mathrm{kg}\cdot m^{-2}\cdot h^{-1}$ under 1 Sun. In the hydrogel system, evaporation is enhanced by regulating the water state within the polymer network: hydrophilic groups and the PVA-rich shell stabilize moderately bound intermediate water through hydrogen-bond interactions, thereby lowering the energy required for vapor generation [86]. These results demonstrate that jellyfish-inspired structural differentiation regulates heat transfer via vortex-mediated convection and hydrogen-bond-governed enthalpy suppression across scales [86,87].

## 5. Thermal Management Strategies Inspired by Arthropoda

**Figure 17:**
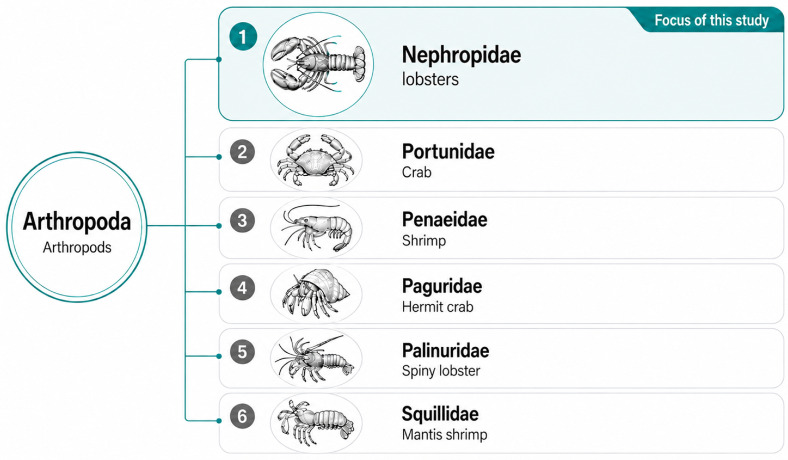
Architectural network of Arthropoda for multifunctional thermal barriers.

Arthropoda is a phylum of ecdysozoan animals defined by a segmented body organization and paired, articulated appendages, all enclosed within a rigid external exoskeleton. This exoskeleton functions as both a protective armor and a primary load-bearing framework, fundamentally shaping arthropod morphology and locomotion [88,89]. The arthropod exoskeleton, or cuticle, is a chitin-based composite structure secreted by a single epidermal cell layer. At the nanoscale, it consists of crystalline α-chitin nanofibrils embedded in a proteinaceous matrix, forming a lightweight yet mechanically robust organic composite. In many arthropod lineages—most prominently crustaceans—the cuticle is further reinforced by inorganic mineral phases such as calcium carbonate or calcium phosphate, resulting in an organic–inorganic hierarchical nanocomposite [89,90,91]. Across diverse arthropod taxa illustrated in Figure 17, the cuticle exhibits a conserved helicoidal or Bouligand architecture generated by the gradual rotation of chitin–protein fiber layers. This structural motif produces pronounced gradients in stiffness and hardness through the cuticle thickness and provides enhanced resistance to fracture, impact, and cyclic mechanical loading. Such a graded composite design represents a unifying biomechanical principle of arthropod exoskeletons rather than a lineage-specific adaptation [89,90]. Because the rigid exoskeleton cannot expand continuously, growth in arthropods is achieved through periodic molting (ecdysis), during which the old cuticle is shed and replaced by a newly secreted, expanded, and subsequently hardened one. This molting-based growth strategy constitutes a fundamental physiological constraint and a defining characteristic of the arthropod body plan [88,92].

### 5.1. Lobster-Inspired Gradient Composite Exoskeletal Systems

Lobsters are marine invertebrates belonging to the phylum Arthropoda and the subphylum Crustacea. Their bodies are protected and mechanically supported by a rigid exoskeleton. This exoskeleton is an organic–inorganic composite that consists of a chitin–protein fibrous matrix reinforced with calcium carbonate minerals. It exhibits a hierarchical architecture, and both the degree of mineralization and the fiber orientation are spatially regulated across different layers. In particular, the multilayered organization, composed of the exocuticle and endocuticle, generates a spatial gradient in hardness and toughness. This gradient enables simultaneous resistance to external impacts and effective dissipation of mechanical energy [93,94]. Such hierarchical energy-dissipation characteristics are not limited to mechanical loading but provide a general structural basis for regulating energy transport across multiple physical domains. Within the exoskeleton, fibers are arranged in planar layers that gradually rotate during stacking, forming a twisted plywood structure. This architecture suppresses crack propagation and distributes fracture energy over multiple length scales. In addition, a well-developed pore canal system forms a honeycomb-like architecture. This system enhances mechanical stability and load distribution while maintaining low density [95]. Collectively, these multilayered, heterogeneous, and composite structural features provide the biological basis for the lobster’s exceptional structural reliability under harsh marine conditions.

**Figure 18:**
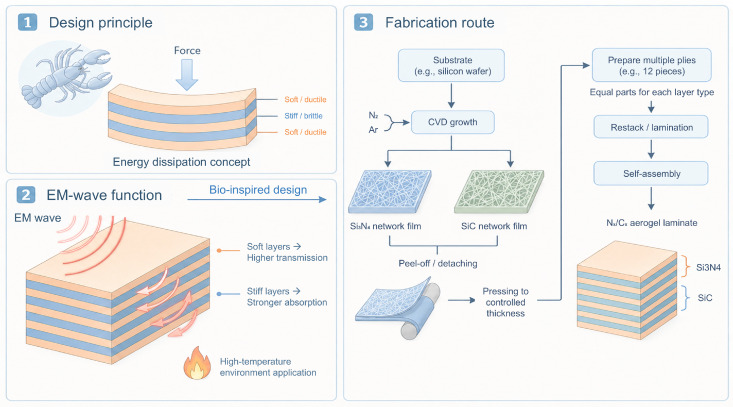
Fabrication of Lobster-Inspired Bouligand Aerogel Laminates for Anisotropic Heat Spreading and Insulation.

A schematic overview of the lobster-inspired design concept and fabrication process is provided in Figure 18. In biomimetic studies inspired by lobsters, the heterogeneous multilayered organization of the exoskeleton and appendages, together with organic–inorganic composite strategies, serves as the primary design motif for functional materials. Cai et al. [96] focused on the ductile–brittle multilayered structure of lobster shells, which dissipates mechanical energy in a stepwise manner during fracture. They translated this mechanism into the design of electromagnetic wave attenuation structures. In their work, ultralight aerogels composed of alternating silicon nitride ($Si_{3}N_{4}$) layers with high electromagnetic transparency and silicon carbide (SiC) layers with strong absorption capability were fabricated. This multilayer design increases the diversity of attenuation pathways compared with single-component ceramic materials [96]. In the resulting multilayer architecture, the surface $Si_{3}N_{4}$ layer improves impedance matching and facilitates the ingress of incident electromagnetic waves. Meanwhile, repeated reflection–absorption–zigzag reflection pathways are generated at the internal $Si_{3}N_{4}$/SiC interfaces. These pathways induce multistage dissipation of electromagnetic energy. As a result, the $Si_{3}N_{4}$/SiC alternately layered aerogel achieved a maximum reflection loss of approximately −45 dB and an effective absorption bandwidth of 8.4 GHz at an ultralow density of ∼$8\;\mathrm{mg}\cdot {\mathrm{cm}}^{-3}$. It also maintained stable electromagnetic absorption performance and thermal insulation properties up to 1000 °C. From a heat-transfer perspective, the ceramic and highly porous $Si_{3}N_{4}$/SiC framework suppresses through-thickness heat transport by interrupting continuous solid-state conduction pathways, while the thermally stable ceramic phases help preserve the multilayer attenuation architecture under thermal exposure [96]. This study experimentally demonstrates that the multilayer energy-dissipation architecture of lobster shells serves as an effective biomimetic motif for structural attenuation design in coupled thermal and electromagnetic environments. Meanwhile, Zhou et al. [97] developed a composite thermal-insulating and flame-retardant material inspired by the organic core–inorganic shell (core–shell) structure of lobster antennae. In this work, a silica shell was formed on the surface of an aramid nanofiber–based aerogel via chemical vapor deposition (CVD). This uniform silica coating allowed the researchers to simultaneously achieve the mechanical flexibility of organic aramid fibers and the thermal and flame resistance of inorganic silica. The resulting core–shell architecture functions as a continuous inorganic barrier that protects the internal aramid network from heat and oxidation. At the same time, it preserves a porous structure that effectively blocks thermal conduction pathways. Mechanistically, this thermal protection originates from the coupled barrier–pore effect: the silica shell limits heat- and oxygen-driven attack on the organic core, while the porous fiber network disrupts continuous solid-state heat-transfer paths. During flame exposure, the silica-rich surface can also form an oxidation-resistant insulating layer, further stabilizing the underlying aramid framework under thermal stress [97]. Consequently, the optimized composite aerogel exhibited a low thermal conductivity of approximately $0.030\;W\cdot m^{-1}\cdot K^{-1}$ and high thermal stability above 530 °C. It also demonstrated excellent flame-retardant performance, with a limiting oxygen index (LOI) of 36.5. These results indicate that the structural duality of lobster antennae provides a viable biomimetic strategy for designing thermal management and protective materials capable of operating under high-temperature and flame-exposed conditions [97].

### 5.2. Crustacean-Shell-Inspired Structural Protection Systems

The shells of crustaceans have been extensively investigated as biological structures that provide outstanding mechanical stability and environmental durability. This performance arises from hierarchical composite architectures composed of chitin–protein–mineral phases, multilayered arrangements, and spatially heterogeneous property gradients. These structural characteristics have primarily been exploited as archetypal models for biomimetic design in applications related to impact absorption, fracture resistance, and lightweight high-stiffness structures. In recent years, a limited number of studies—mainly focusing on lobster-inspired systems—have also explored their potential for industrial implementation. In contrast, beyond these isolated cases, engineering studies that translate or replicate crustacean shell architectures across broader taxa, such as shrimps, crabs, and horseshoe crabs, for the purpose of enhancing thermal management functions—including heat conduction, thermal insulation, heat dissipation, thermal stability, or temperature regulation—are still scarce. Although biological observations regarding the thermal environmental adaptation and structural heat tolerance of crustacean shells do exist, systematic extensions of these insights into the design of functional thermal management materials or systems have not yet been fully realized. This trend indicates that crustacean-inspired biomimetic research has remained heavily biased toward mechanical functionality, while simultaneously revealing a distinct research gap in the context of thermal management–oriented biomimetic design.

## 6. Thermal Management Strategies Inspired by Echinodermata

**Figure 19:**
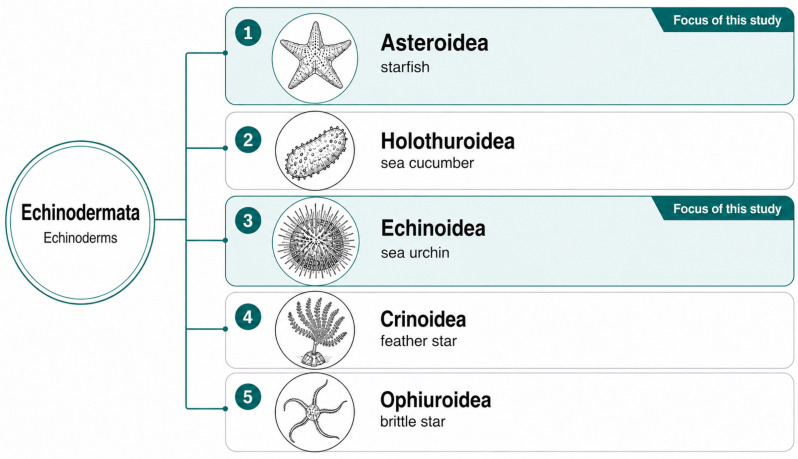
Comprehensive network of Echinodermata for enhanced thermal energy storage.

Echinodermata is a phylum of exclusively marine deuterostome invertebrates defined by a distinctive integration of developmental, skeletal, and functional traits. Extant echinoderms comprise five major classes and uniquely exhibit adult pentaradial symmetry that is secondarily derived from a bilaterally symmetrical larval ancestor [98,99]. A central defining feature is the water vascular system, a hydrocoel-derived hydraulic network that underlies locomotion, feeding, adhesion, and gas exchange. This fluid-based actuation system is conserved across crown-group echinoderms and distinguishes them fundamentally from other metazoan phyla relying primarily on muscular or ciliary mechanisms [98,99]. The internal classification of Echinodermata is schematically illustrated in Figure 19. Echinoderms are further characterized by a calcitic endoskeleton composed of stereom, a porous three-dimensional microarchitecture that constitutes the principal synapomorphy of the phylum. Genetically regulated stereom enables intimate mechanical coupling with mutable collagenous tissues, allowing reversible modulation of stiffness and mechanical performance. Fossil evidence demonstrates that this biomineralization strategy was already established in Cambrian echinoderms [100]. Phylogenetically, echinoderms form the sister group to hemichordates within Ambulacraria. Molecular and morphological data indicate that the ancestral echinoderm possessed a bilaterally symmetrical adult body plan, with pentaradial organization arising secondarily through torsion and internal reorganization during metamorphosis [99]. Early echinoderms exhibited high morphological disparity, and quantitative analyses show that modern body plans were progressively established rather than fixed at the origin of the phylum [101]. Collectively, Echinodermata is defined by the integrated coupling of hydraulic actuation, stereom-based biomineralization, and mechanically adaptive connective tissues, rendering the phylum a distinctive biological system and a valuable source of biomimetic inspiration.

### 6.1. Starfish-Inspired Thermo-Responsive Structural Regulation Systems

Sea stars (Class Asteroidea) are dorsoventrally flattened echinoderms characterized by a pentaradial body plan in which multiple rays radiate from a central disc, bearing ventral ambulacral grooves with tube feet [102]. All extant taxa belong to the post-Paleozoic Neoasteroidea and are morphologically distinguished by the structure of the ambulacral column [102,103]. The body wall encloses an articulated endoskeleton composed of numerous calcareous ossicles embedded within mutable collagenous tissue. Each ossicle consists of a porous stereom architecture formed primarily of magnesium-enriched calcite [104]. The spatial organization of ambulacral, adambulacral, marginal, and aboral ossicular systems generates a mechanically integrated yet flexible skeletal network that enables coordinated ray motion without rigid fusion [103,105]. Accordingly, sea stars may be defined as radially organized, stereom-based ossicular endoskeletal organisms in which modular calcitic elements and mutable connective tissues collectively produce a flexible load-bearing biomechanical system.

**Figure 20:**
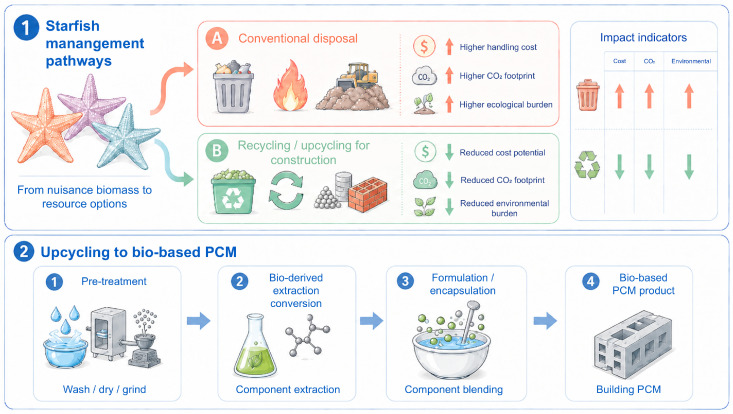
Upcycling of starfish biomass into bio-based PCMs for enhanced latent heat storage.

In the study by Yun et al. [106], the porous calcium-carbonate skeleton of starfish was employed as a supporting scaffold for shape-stabilized phase-change materials (PCMs) to enhance thermal energy storage performance. The authors experimentally demonstrated that the micrometer-scale pore network within the starfish skeleton effectively confines liquid-phase PCMs, thereby suppressing leakage during phase transitions and enabling stable latent heat storage. Mechanistically, the calcium-carbonate-based starfish skeleton functions as a capillary-retention scaffold: when the PCM melts, its liquid phase is held within the pore and closed-cell network by capillary action and physical confinement, allowing latent heat to be stored and released while minimizing outward leakage. The starfish-derived PCM conversion route is outlined in Figure 20. The resulting system exhibited a latent heat capacity of up to ∼$57.66\;J\cdot g^{-1}$ and thermal stability below 150 °C, illustrating a reinterpretation of the starfish skeleton as a functional thermal platform for controlled heat storage and release [106]. Meanwhile, Raman et al. [107] designed a 4D morphing structure inspired by the ossicle–MCT–dermis composite architecture of starfish skeletons, in which stiffness and shape are reversibly switched by thermal stimuli. In this work, heat does not merely serve as an environmental variable or load condition but acts as an active control parameter that governs the mechanical state of the structure. The thermoplastic mesh maintains high stiffness and shape fixation below the glass-transition temperature (Tg), while above Tg it undergoes rapid softening, enabling deformation and reprogramming. The thermal cycle operates as a reversible mechanical-state switch: Heating softens the thermoplastic mesh and permits mechanically driven reconfiguration, whereas subsequent cooling restores rigidity and fixes the newly imposed shape without sustained actuation. This approach directly translates the biological mechanism by which MCT modulates bonding states to fix or relax posture in starfish into a heat-responsive mechanical state-switching system [107]. In addition, Ma et al. [108] numerically investigated the effects of a noncircular, starfish-inspired cylindrical geometry on fluid flow and heat-transfer characteristics. Their analysis showed that protrusions (tubercles) derived from starfish arm morphologies regulate vortex generation and boundary-layer separation, leading—under specific Reynolds numbers and angles of attack—to an increase in the average Nusselt number (Nu), i.e., enhanced convective heat-transfer performance. In this convective mechanism, the starfish-like protrusions alter flow separation and vortex evolution, thereby modifying the distribution of the thermal boundary layer and local temperature gradients around the heated cylinder; in this way, geometry becomes a passive control factor for convective heat-transfer regulation. This study extends the direct morphological imitation of starfish into a passive thermal management strategy for improved heat dissipation and cooling efficiency [108]. Collectively, these starfish-inspired studies demonstrate that heat can act as a functional design parameter rather than a passive environmental variable. This perspective establishes a new paradigm for thermal functionality that integrates energy storage, mechanical switching, and fluid–thermal regulation, surpassing traditional heat-conduction- and heat-dissipation-centric paradigms.

### 6.2. Sea-Urchin-Inspired Radial Thermal Transport Systems

Sea urchins are a major clade of echinoderms characterized by a rigid, globular to flattened endoskeleton composed of interlocking plates of high-magnesium calcite arranged in a pentaradial pattern. The test is subdivided into ambulacral and interambulacral columns and bears movable spines articulated by tubercles, providing both mechanical protection and functional interaction with the environment [109]. The calcitic skeleton of echinoids exhibits a distinctive porous microarchitecture, often described as stereom, which confers high stiffness-to-weight ratios and enhanced resistance to bending and buckling through structural rather than purely material optimization [110]. Internally, echinoids possess a conserved axial complex that integrates coelomic and haemal systems along the oral–aboral axis, while notable morphological divergence between regular and irregular echinoids reflects evolutionary reorganization associated with locomotion, feeding strategy, and sediment interaction [111]. Owing to their robust mineralized skeleton and long evolutionary history, echinoids have left an exceptionally rich fossil record, enabling detailed phylogenetic reconstruction and classification based primarily on skeletal morphology across both extant and extinct taxa [109].

**Figure 21:**
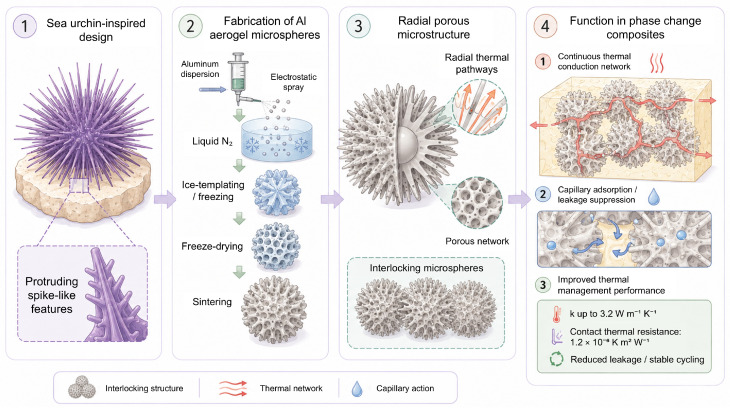
TPMS-based design of sea urchin-inspired MFPCMs for uniform heat flux dissipation.

Zhang et al. [112] fabricated aluminum aerogel microspheres that mimic the spine-like radial structure of sea urchins and used them as thermally conductive fillers in paraffin-based PCM composites. The sea-urchin-inspired aerogels promote mechanical interlocking between fillers through radially extending microstructures and surface protrusions, thereby forming continuous heat-transfer networks within the composite. From a heat-transfer perspective, the radial spike structures transform isolated particle contacts into interlocked filler–filler contacts, allowing heat to pass through a more continuous Al network rather than through discontinuous contacts across the low-conductivity paraffin matrix. Consequently, the composite achieved a thermal conductivity of $3.2\;W\cdot m^{-1}\cdot K^{-1}$ at a filler loading of 43.9 vol%, while interfacial thermal resistance was reduced to $1.2\times 10^{-6}\;K\cdot m^{2}\cdot W^{-1}$, and capillary effects from the porous aerogel structure effectively suppressed PCM leakage during repeated thermal cycling. During melting, capillary adsorption within the porous aerogel retains liquid paraffin around the Al framework, limiting matrix migration and preventing leakage from interrupting the conductive network [112]. Tian et al. [113] highlighted the similarity between the microporous arrangement in sea urchin skeletal cross-sections and primitive triply periodic minimal surface (TPMS) structures and, based on this analogy, designed PCM composites incorporating TPMS-based metallic foam skeletons. Compared with conventional lattice-type metal foams, the the Primitive TPMS-based frameworks provided more continuous and compact heat-transfer pathways, shortening the melting time by approximately 20% and markedly enhancing the effective thermal conductivity, as confirmed by experiments and numerical simulations. Mechanistically, the TPMS skeleton reduces the spatial variation of equivalent thermal resistance by replacing a conventional strut-based lattice with a continuous minimal-surface framework, which promotes more uniform heat spreading and faster advancement of the PCM melting front. These findings show that the intrinsic spatial continuity and connectivity of sea urchin–inspired skeletal architectures are key structural factors that accelerate thermal response in phase-change-based thermal energy storage systems [113]. Furthermore, several studies have adopted sea-urchin-inspired hollow structures engineered for light absorption and photothermal conversion, thereby simultaneously improving photothermal conversion efficiency and latent heat storage performance. This approach extends the role of sea-urchin-inspired architectures from pure heat-transfer enhancement to integrated energy conversion–storage systems, thereby broadening the application space of sea-urchin-based biomimetic thermal management technologies. Figure 21 illustrates the pentaradial skeletal organization of an echinoid, highlighting the interlocking high-magnesium calcite plates and the articulated spine-bearing tubercles that define its external skeleton. Overall, sea-urchin-inspired thermal management studies demonstrate that structural redesign of heat-transfer pathways using radial skeletal architectures and continuous porous networks can enhance latent heat storage rate, thermal conductivity, and thermal stability at the same time. These results indicate that the structural characteristics of sea urchin skeletons function not only as morphological motifs but also as core design principles governing the performance of thermal energy storage and management systems.

## 7. Challenges and Limitations

To strengthen the analytical basis of this section, the major strengths and remaining challenges of the reviewed studies are cross-referenced with the representative engineering outcomes summarized in Table 1, Table 2, Table 3, Table 4 and Table 5. Rather than presenting these challenges as general weaknesses of marine-invertebrate-inspired thermal management, Table 6 organizes them as translation-oriented issues that should be addressed to improve comparability, durability, scalability, and system-level integration. This approach reflects the multidisciplinary nature of the reviewed systems, which involve distinct thermal-management functions such as radiation control, convective heat transfer, interfacial thermal transport, thermal insulation, evaporation, and phase-change heat storage.

### 7.1. Structural Complexity and Manufacturability

Despite increasing research interest in marine invertebrate-inspired thermal management strategies, several critical challenges remain before these concepts can be translated into practical engineering systems. One major limitation arises from the intrinsic geometric and hierarchical complexity of marine invertebrate structures. Porous skeletons, multiscale lamellar architectures, spiral geometries, and dynamically reconfigurable interfaces—while functionally advantageous in biological systems—often require sophisticated fabrication routes such as freeze casting, templating, multistep self-assembly, or high-resolution additive manufacturing. Consequently, many reported architectures remain confined to laboratory-scale demonstrations, and the scalability, reproducibility, and cost efficiency required for industrial thermal management applications have not yet been sufficiently validated.

### 7.2. Long-Term Thermal and Environmental Stability

Another major challenge concerns the long-term stability of bioinspired thermal materials under realistic operating conditions. While many studies report promising thermal performance under steady-state or short-duration testing, practical applications typically involve cyclic thermal loading, mechanical deformation, and exposure to harsh environments, including high humidity, salinity, pressure fluctuations, or combined thermal–chemical stress. For porous, gel-based, or polymer-rich systems, degradation mechanisms such as pore collapse, interfacial delamination, material fatigue, or leakage of phase change materials remain insufficiently characterized, leading to uncertainties in long-term reliability.

### 7.3. Limited Integration of Coupled Heat-Transfer Mechanisms

In biological systems, thermal regulation arises from tightly coupled conduction, convection, radiation, and interfacial heat transfer processes. In contrast, many bioinspired engineering studies focus on a single dominant heat-transfer mode, such as enhancing thermal conductivity, controlling radiative emissivity, or improving convective heat transfer. This reductionist approach may overlook system-level interactions and, in some cases, limit achievable performance improvements. Integrated demonstrations that simultaneously consider multiple heat-transfer mechanisms within a unified material, device, or system architecture remain relatively limited.

### 7.4. Quantitative Comparability and Lack of Standardization

A further limitation lies in the lack of standardized evaluation protocols. Thermal metrics such as thermal conductivity, radiative cooling performance, temperature reduction, or heat flux are often measured under different boundary conditions, sample geometries, and environmental settings. This variability complicates direct comparison across studies and hinders objective benchmarking against conventional thermal management technologies. In addition, only a limited number of studies evaluate bioinspired designs alongside industrial reference materials under identical testing conditions.

### 7.5. Biological Abstraction and Functional Oversimplification

Finally, a conceptual limitation arises from the abstraction of biological inspiration. Many bioinspired designs extract isolated structural motifs or material features from marine invertebrates while neglecting the dynamic, adaptive, and multifunctional nature of the original biological systems. As a result, engineered analogs may replicate morphological resemblance without fully capturing underlying regulatory mechanisms such as adaptive response, environmental feedback, or self-repair. Bridging this gap will require closer integration of biological insight, physical modeling, and engineering validation.

### 7.6. Outlook

Addressing these challenges will require advances in scalable manufacturing methods, systematic durability testing under coupled environmental stressors, and system-level integration of multiple heat-transfer mechanisms. Establishing standardized evaluation frameworks and improving alignment between biological function and engineering abstraction will be essential for translating marine invertebrate-inspired thermal management strategies into deployable technologies.

## 8. Future Perspectives

### 8.1. Toward Scalable and Manufacturable Bioinspired Architectures

To address challenges associated with structural complexity and manufacturability, future research should prioritize bioinspired architectures that balance functional fidelity with fabrication feasibility. Rather than directly replicating highly intricate biological geometries, abstraction of key structural principles—such as hierarchical porosity, directional anisotropy, or modular repetition—may enable scalable implementations using established manufacturing routes. Advances in additive manufacturing, roll-to-roll processing, and template-free self-assembly are expected to play a critical role in translating marine invertebrate-inspired designs from laboratory-scale demonstrations to large-area or high-throughput thermal management components.

### 8.2. Enhancing Durability Under Coupled Thermal and Environmental Stressors

Improving long-term thermal and environmental stability requires systematic evaluation under conditions that more closely reflect real-world operation. Future studies should incorporate cyclic thermal loading, mechanical deformation, humidity, salinity, and pressure variations into durability testing protocols. In addition, coupling in situ characterization techniques with accelerated aging tests may help elucidate degradation mechanisms in porous, polymer-based, or PCM-containing systems. Such efforts will be essential for establishing performance reliability over extended service lifetimes.

### 8.3. System-Level Integration of Multi-Mode Heat Transfer

Future bioinspired thermal management strategies should move beyond isolated enhancement of individual heat-transfer modes toward integrated system-level designs. Drawing inspiration from marine invertebrates, simultaneous consideration of conduction, convection, radiation, and interfacial heat transfer within a unified architecture may unlock synergistic performance gains. Coupling experimental validation with multiphysics modeling will be particularly important for optimizing interactions between thermal transport, fluid flow, and mechanical response at the device and system scales.

### 8.4. Establishing Standardized Evaluation and Benchmarking Frameworks

To improve quantitative comparability across studies, standardized evaluation frameworks for bioinspired thermal materials and systems should be developed. Defining common testing conditions, boundary parameters, and reporting metrics will facilitate objective benchmarking against conventional thermal management technologies. Moreover, incorporating industrial reference materials and application-relevant geometries into experimental studies will help clarify the practical advantages and limitations of marine invertebrate-inspired designs.

### 8.5. Bridging Biological Function and Engineering Abstraction

Future progress will also depend on narrowing the gap between biological inspiration and engineering implementation. Rather than focusing solely on morphological resemblance, bioinspired thermal designs should aim to capture functional principles such as adaptability, environmental responsiveness, and multifunctionality. Closer collaboration among biologists, materials scientists, and thermal engineers will be essential for translating dynamic biological strategies into robust, controllable, and application-ready thermal management systems.

## 9. Conclusions

This review systematically examined thermal management strategies inspired by five major marine invertebrate phyla—Mollusca, Porifera, Cnidaria, Arthropoda, and Echinodermata—and organized the corresponding engineering literature into three implementation levels: bio-inspired functional materials, structural architectures, and integrated thermal management systems. By consolidating dispersed case studies within a unified comparative framework, several cross-cutting patterns and insights have emerged that were not apparent from individual studies alone.

A first key observation is the uneven distribution of research effort across biological lineages and heat-transfer modes. Among the five phyla surveyed, Mollusca has generated the most diverse portfolio of thermal management strategies, spanning dynamic infrared modulation (Cephalopoda), directional thermal conduction (Gastropoda), and wet-stable interfacial thermal transport (Bivalvia). In contrast, Arthropoda-inspired research remains heavily concentrated on mechanical functionality, with thermal translation largely confined to lobster-derived gradient composites, revealing a pronounced and exploitable research gap. Porifera-inspired work, while limited in volume, introduces a conceptually distinct paradigm—passive, structure-induced flow organization for convection-driven heat exchange without external energy input—that has no direct analogue among the other phyla reviewed.

A second finding concerns the relationship between the biological mechanism and the dominant heat-transfer mode. Radiation-based thermal regulation, including infrared emissivity modulation and solar absorptance control, is predominantly reported in cephalopod- and coral-inspired systems, where chromatophore-like multilayer architectures or multiscale porous skeletons govern optical-thermal interactions. Conduction-based strategies, particularly directional thermal pathway engineering, are concentrated in gastropod (nacre) and echinoderm (sea urchin) systems that exploit hierarchical lamellar or radial skeletal geometries. Convection-based and phase-change-coupled strategies appear most prominently in nautilus-inspired spiral flow designs, jellyfish-inspired hollow evaporators, and starfish-/sea-urchin-derived PCM scaffolds. This mapping reveals that no single biological lineage addresses all three heat-transfer modes simultaneously, suggesting that future high-performance thermal systems may benefit from hybridizing design principles across phylogenetically distinct templates—for instance, combining cephalopod-derived adaptive emissivity control with echinoderm-derived latent heat storage architectures.

A third insight relates to the gap between reported performance metrics and practical deployment readiness. While several studies demonstrate impressive quantitative results—such as cuttlebone-inspired MXene aerogels achieving thermal conductivities of 0.021–0.025 $W\cdot m^{-1}\cdot K^{-1}$ (comparable to still air), squid-inspired composites sustaining optical–thermal modulation over 25,000 deformation cycles, and coral-inspired solar absorber coatings maintaining 97.39% absorptance after 3000 h at 800 °C—the majority of these demonstrations remain at the coupon or laboratory scale. Scalable fabrication routes, standardized durability protocols under coupled thermal-mechanical-chemical stressors, and direct benchmarking against incumbent industrial materials are largely absent from the current literature. Bridging this translation gap represents the most critical near-term challenge for the field.

Finally, this review identifies a recurring conceptual limitation in how biological inspiration is abstracted. Many engineering studies sometimes replicate static morphological features-such as pore geometry, lamellar stacking, or spiral curvature-while omitting the dynamic, responsive, and self-regulating characteristics that make biological thermal management truly distinctive. Octopus-inspired adaptive interfaces and starfish-inspired thermally triggered stiffness modulation represent early exceptions, but the broader integration of stimulus-responsiveness into bioinspired thermal materials remains nascent. Advancing from morphological mimicry toward functional mimicry—capturing not just structure but adaptability—will be essential for realizing the full potential of marine invertebrate-inspired design.

In summary, while significant challenges remain in manufacturability, long-term reliability, multi-mode integration, and standardized evaluation, the diversity and sophistication of marine invertebrate-inspired thermal strategies reviewed here establish a compelling foundation for next-generation thermal technologies. Realizing this potential will require not only continued advances in materials processing and systems engineering, but also deeper interdisciplinary collaboration that faithfully translates the adaptive, multifunctional logic of marine invertebrate biology into deployable engineering solutions.

## Figures and Tables

**Table 1:** Representative Mollusca-inspired thermal-management systems and thermal-management-relevant engineering outcomes.

Structure	Implementation	Mechanism	Indicator	Application	Refs.
Squid pigment cells	Optical multilayer	Radiation	ΔT: −18 to +4 °C	Camouflage	[21]
Cuttlebone lattice	MXene aerogel	Insulation	k: 0.021–0.025 W·m^−1^·K^−1^	Thermal shielding	[25]
Octopus papillae	Microchannel film	Convection	IR: −70%	Surface cooling	[30]
Octopus suckers	Adhesive interface	Interface	h: >2×	TIMs	[31]
Nautilus shell	Spiral flow field	Convection	P_peak_: +21.5%	PEMFCs	[35]
Abalone nacre	BNNS/epoxy laminate	Conduction	k(In-plane): enhanced	Composites	[46]
Spiral shell	Ribbed structure	Dissipation	Crack delay: +28%	Protection	[51]
Mussel byssus	PDMS/EGaIn	Interface	k: 6.9 W·m^−1^·K^−1^	TIMs	[59]
Mussel coating	Radiative multilayer	Radiation	ΔT: −5.3 °C	Cooling coating	[59]

**Table 2:** Representative Porifera-inspired thermal-management systems and thermal-management-relevant engineering outcomes.

Structure	Implementation	Mechanism	Indicator	Application	Refs.
Helical ridges	Helical lattice	Convection	〈uz〉>0	Passive ventilation	[69]
Central cavity	Flow channel	Convection	Outflow/inflow: ∼37%	Heat/mass exchange	[69]
Sponge skeleton	Graphene aerogel	Porous transport	Height recovery: 99.9%	Thermal diffusion	[64]

**Table 3:** Representative Cnidaria-inspired thermal-management systems and thermal-management-relevant engineering outcomes.

Structure	Implementation	Mechanism	Indicator	Application	Refs.
Coral skeleton	TiO_2_ coating	Radiation	$\alpha_{\mathrm{solar}}$: 97.39%	CST receiver	[80]
Coral porous network	Cooling film	Radiation	8–13 μm emission	Radiative cooling	[81]
Jellyfish umbrella	Hollow evaporator	Evaporation	$\eta_{\mathrm{evap}}$: 108.9%	Solar evaporation	[85]
Jellyfish bell	Heat-sink fins	Convection	TPF: +20.06%	Microchannel cooling	[87]
Jellyfish head	Hydrogel evaporator	Evaporation	Rate: 1.90 kg·m^−2^·h^−1^	Interfacial evaporation	[86]

**Table 4:** Representative lobster-inspired thermal-management systems and thermal-management-relevant engineering outcomes.

Structure	Implementation	Mechanism	Indicator	Application	Refs.
Lobster exoskeleton	Si_3_N_4_/SiC aerogel	Insulation	T_stable_: 1000 °C	Thermal protection	[96]
Lobster antenna	Aramid@silica aerogel	Insulation	k: ∼0.030 W·m^−1^·K^−1^	Flame protection	[97]

**Table 5:** Representative Echinodermata-inspired thermal-management systems and thermal-management-relevant engineering outcomes.

Structure	Implementation	Mechanism	Indicator	Application	Refs.
Starfish skeleton	PCM scaffold	Phase change	ΔH: 57.66 J·g^−1^	Heat storage	[106]
Starfish ossicles	4D structure	Thermal switching	Tg-triggered	Morphing system	[107]
Starfish arms	Noncircular cylinder	Convection	Nu: enhanced	Passive cooling	[108]
Sea urchin spines	Aerogel/PCM	Conduction	k: 3.2 W·m^−1^·K^−1^	PCM transfer	[112]
Sea urchin skeleton	TPMS foam/PCM	Phase change	Melting: >20% faster	Heat storage	[113]
Sea urchin cavity	Photothermal PCM	Photothermal	Conversion: enhanced	Solar storage	[113]

**Table 6:** Cross-referenced strengths and remaining challenges for practical translation of marine-invertebrate-inspired thermal-management systems.

Study Group	Representative Strengths	Remaining Challenges for Practical Translation	Related Evidence	Representative Refs.
Mollusca-inspired systems	Broad functional diversity across radiation control, insulation, convection, interfacial thermal transport, conduction, and structural dissipation	Standardized benchmarking across different applications would improve cross-study comparability	Table 1; Section 7.3 and Section 7.4	[21,25,30,31,35,46,51,59]
Porifera-inspired systems	Hierarchical porous, helical, and cavity-containing architectures for ventilation, flow guidance, heat/mass exchange, and porous transport	Further direct thermal-performance validation would clarify their relevance to device-level heat-transfer applications	Table 2; Section 7.1 and Section 7.4	[69,70]
Cnidaria-inspired systems	Radiation-control, radiative-cooling, evaporation, and microchannel-cooling functions	Unified comparison across radiation, evaporation, and convection modes would strengthen quantitative assessment	Table 3; Section 7.3 and Section 7.4	[80,81,85,86,87]
Lobster-inspired systems	Effective models for thermal insulation and flame/thermal protection	Broader validation under coupled thermal, mechanical, and environmental stresses would support long-term reliability	Table 4; Section 7.2 and Section 7.4	[96,97]
Echinodermata-inspired systems	Diverse strategies for phase-change heat storage, thermal switching, passive cooling, PCM heat-transfer enhancement, and solar thermal storage	Additional cycling, leakage-resistance, structural-stability, and system-level validation would support practical integration	Table 5; Section 7.2, Section 7.3 and Section 7.4	[106,107,108,112,113]

## Data Availability

The datasets generated during the current study are available from the corresponding author on reasonable request.

## Abbreviations

The following abbreviations are used in this manuscript:

PCM	Phase-change material
PCMs	Phase-change materials
IR	Infrared
Tg	Glass-transition temperature
Nu	Nusselt number
R_solar	Solar reflectance
ϵ_LWIR	Long-wave infrared emissivity
Re	Reynolds number
PEMFC	Proton exchange membrane fuel cell
CFD	Computational fluid dynamics
rDNA	Ribosomal DNA
COI	Cytochrome c oxidase subunit I
MXene	Titanium carbide MXene, Ti_3_C_2_T_*x*_
AFM	Atomic force microscopy
PDMS	Polydimethylsiloxane
MAPs	Mussel adhesive proteins
EGaIn	Eutectic gallium–indium
TOCN	Tempo-oxidized cellulose nanofiber
BNNS	Boron nitride nanosheet
MCT	Mutable connective tissue
